# Multi-criteria of PV solar site selection problem using GIS-intuitionistic fuzzy based approach in Erzurum province/Turkey

**DOI:** 10.1038/s41598-021-84257-y

**Published:** 2021-03-03

**Authors:** Seda Türk, Ahmet Koç, Gökhan Şahin

**Affiliations:** 1 Engineering Faculty, Industrial Engineering Department, Igdir University, Igdir, Turkey; 2 Vocational School of Technical Sciences, Park and Garden Plants Department, Dicle University, Diyarbakır, Turkey; 3 Engineering Faculty, Department of Electric Electronic Engineering, Igdir University, Igdir, Turkey

**Keywords:** Environmental impact, Environmental sciences, Energy science and technology, Engineering

## Abstract

Renewable energy sources have been placed as the key to facilitating to provide source of electricity generation. Solar power is one of the most preferable one among renewable energy sources due to the easy to generate in suitable environment. However, there are concerns with the location of solar power plants installation which causes low efficiency and ineffective use. Hence, determining the location for the usage of solar power sources is critical to mitigating those concerns. In addition, Turkey has been focused on investments on sustainable renewable energy sources and there are few studies which work on cities to reveal potential sources. In this study, GIS and intuitionistic fuzzy set based multi-criteria decision-making method is proposed for determining the most suitable areas for solar energy power plant potential site selection in Erzurum province, Turkey. Firstly, a solar energy power plant potential site selection map is made using a GIS program along with considering ecological risks and ecological criteria. Secondly, 20 districts of Erzurum are investigated in terms of 10 criteria (slope, aspect, solar irradiation, land use, wind speed, air temperature, air pressure, air humidity, land surface temperature and transmission line) using intuitionistic fuzzy sets. In these parameters the first time we looked the land use surface. The land use surface is affected the efficiency of the solar power plant. Finally, the comparisons of two methods are done to check consistency of results obtained. The results indicate that both approaches achieve same areas as the most suitable locations for solar power plants installations for Erzurum province in Turkey. The aim of this work is first to investigate possible locations for solar power plant installation using a mapping method, GIS, and then, Intuitionistic Fuzzy is applied to the problem to obtain optimum areas for solar energy. Also, more accurate results are provided comparing results of two methods, GIS and Intuitionistic Fuzzy. The results expose that 25,065.3 km^2^ for solar power plant suitable for solar power plan installation.

## Introduction

The constant growth on the world population is interaction with an increase on energy supply^1–3^. According to Pimentel et al., there has been a significant reduction that cannot be ignored on the fossil resources reserves and in 30–40 years, these reserves will be unable to meet the needs of the world^4^. The fact that excessive consumption of the limited fossil resources has led people to turn to alternative energy sources such as wind, solar energy. Indeed, demand for electricity-based energy has increased steadily while the development on the technology has been raised for a hundred years. This situation has a significant impact on the use of electricity and the amount of usage has doubled every 14.5 year period^5,6^.

As a matter of fact, the inevitable source of electricity generation is determined as fossil fuels which have the biggest share with 60%^7^. The upsurge in the usage of fossil fuels has essential effects not only on the environment but also climate^8,9^. Based on the report of the Union, in order to control the increase on environmental problems and deterioration, and to alleviate climate change, the usage of renewable energy sources as an alternative energy sources has been need to focus^10^. In addition, the policies of countries have been changed with respect to this report. With the previous policies approaching the irreversible point in natural life and environmental degradation to ensure a sustainable environment, economic growth policies have started to be created in a sustainable development perspective, taking care of the environment and natural life^11,12^. In order to make these policies applicable, well-analysed lands is necessary in terms of revealing renewable energy sources. The advancing technology also provides convenience to these analyses nowadays. Package programs working with geographic information systems (GIS) and remote sensing infrastructures are especially for a great support in the installation of renewable energy power plants.

In Turkey, the demand of energy along with population growth has been increased day by day. The improvement on economy and living standards has also increased the energy needed. Solar energy systems which has been playing a significant role on dealing with energy problems, one of these alternative energy sources, has been spread^13,14^. Eastern Anatolia Region which has 2664 h of solar irradiation duration and annual 1365 kWh/m^2^ radiation is considered as an important region with placing 3rd out of 7 regions in Turkey in terms of potential of the solar energy^15^. In addition, the city which has the largest area in the Eastern Anatolia Region is Erzurum with an approximate 25.355 km^2^ and it has topographically active structures and wide plains. The fact that the area of Erzurum is so high enables the establishment of many solar power plants of Erzurum on a provincial scale. As a matter of fact, Erzurum^16^ which hosted a single installed solar power plant with the first solar power plant license issued in 2014, Erzurum province is hosted a 33 large and small solar power plants year of 2018^17^. In this study, we address the issue of determining the desirable locations for a solar energy power plant in the provincial scale by taking into account ecological risks and ecological criteria in a way to ensure the least destruction on the natural balance of Erzurum province.

In literature, in the study conducted by^18^ to determine the solar energy potential of the Knjazevac region in the east of Serbia, 4 criteria were determined: climate, topography, solstice and land use. 11 criteria were considered in the study conducted by^19^ to determine the areas where GES will be established in Iran. Since the advantages of the criteria are not certain, Fuzzy Analytical Hierarchy Process (BAHS) was used in weighting and a GES compliance map for Iran was created in the GIS environment. In addition, the most suitable regions for GES were determined by taking 1057 regions of the country into consideration. As a result of the study conducted by^20^ using AHS and GIS for the whole of Saudi Arabia, it was revealed that the most suitable areas for GES are the northern and north-western parts of the country. It has been understood that the distance to the roads, energy transmission lines and settlements plays a role in the determination of these areas. In the study conducted by^21^, the areas where GES will be established in Khuzestan city of Iran were determined using BAHS and GIS. As a result of the study, it was understood that the city of Khuzestan has a great potential. Even in the worst scenario, it has been calculated that the energy to be produced in this region will be 1.75 times the energy produced in all of Iran in 2013. At the same time, it is thought that the stations to be established in the south and southwest of the region will be effective against desertification in this region. In the study in^22^, 4 main criteria and 8 sub-criteria were determined, namely climate, water resources, location and topography, in order to determine the areas will be established in the east of Morocco has been created. In the literature review, it was determined that AHS and BAHS methods are widely used in the creation of the GIS compatibility map. In the literature review, it was determined that AHS and BAHS methods are widely used in the creation of the GIS compatibility map. In this study, it was aimed to compare the use of classical and Intuitionistic Fuzzy Based Approach in creating a suitability map. For this purpose, the analysis results obtained when using both methods were examined and it was aimed to discuss the advantages and disadvantages of the methods compared to each other.

**Figure 1:**
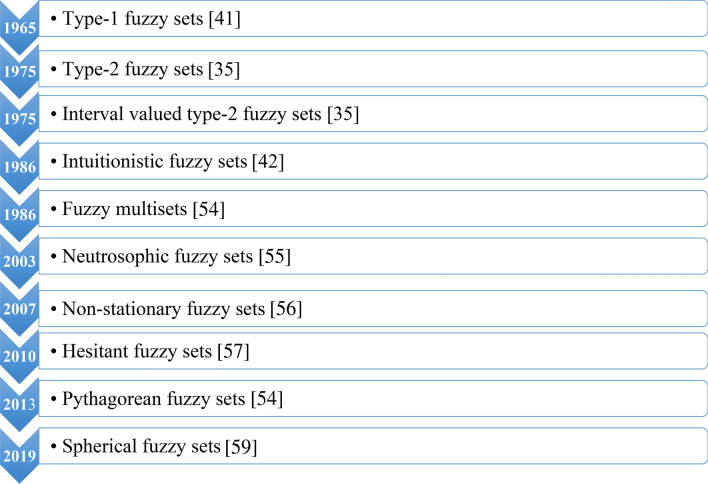
Extensions of fuzzy sets^34,35,41,42,54–59^.

There are various previous studies tackling the problem of optimal location selection for a power plant installation. One of the most convenient methods is mapping techniques and these techniques and the developing technology have been adapted into a system namely GIS. Based on the literature, the integration of renewable energy into the GIS base worldwide has been done^23–27^. At the same time in the solar power plant^28,29^, wind power plants^30–32^ and hybrid power plant in choice of place^33^ made use of GIS and remote sensing infrastructure systems to make use of areas suitable for ecological criteria in Fig. 1.

In addition, location selection problems for solar power plants are not based on precise measures, but often on vague and imprecise terms. In order to deal with uncertainties, Zadeh^35^ introduced the fuzzy set theory which allows dealing with linguistic assessments precisely. Fuzzy set theory has been extended to a number of types as shown in Fig. 1. The scientific literature shows that each type has been applied to many fields and few of them has been used as a crucial part of the solution methods for installation of renewable energy plants for a decade. Based on the literature, generally type-1 fuzzy sets have been applied to either directly or combining with other multi-criteria decision making methods such as the analytic hierarchy process (AHP), VIKOR on solar power plant site selection problems. Yousefi et al.^36^ employed a fuzzy-GIS model to determine appropriate areas for solar power plants installation in Markazi, Iran with respect to technical, economic and environmental criteria. In addition, Zoghi et al.^37^ stated that renewable energy will become an indispensable place for countries and Iran is located a desirable place as a solar energy source. For this reason, in their work, a fuzzy model is used to identify criteria and then Isfahan province in Iran is examined in terms of criteria determined by the fuzzy model proposed.

There are also some methodologies proposed combining fuzzy methods with another method in solar energy decision making problems. For instance, Wiguna et al.^38^ addressed a fuzzy AHP and PROMETHEE approach for solar farm site selection problem in Bali. Kahraman and Otay pointed out a Z-fuzzy based AHP method which provides a better representation than ordinary fuzzy numbers and it is applied to a solar PV power plant location selection^34^. In the work of Solangi et al.^39^, a fuzzy-VIKOR technique is applied to assessment of 14 cities in Pakistan to find out the most suitable cities for the installation of solar PV power plants with considering several criteria such as economic, environmental. According to the literature, few studies exist on extensions of fuzzy sets because of their computational complexity and the majority of the researchers have focused on type-1 fuzzy models. However, in this study, an intuitionistic fuzzy based approach is proposed to deal with a site selection problem of solar energy power plant installation.

## Background

This section introduces the fundamentals of the techniques used in this work including GIS as a mapping technique and intuitionistic fuzzy sets.

### Mapping technique, GIS

Geographical Information Systems differ from other information systems in terms of the data they contain and the properties of this data. The ability to address the location of objects or events (phenomena) on earth is characteristic of such data. For this reason, the locations of objects or events and their relationships with each other can be visualized and this visualization is called a "map". Objects in the real world (house, road, mountain, etc.) are summarized according to the specified criteria; a topographic landscape model is created and stored in GIS as point, line, area or volume. Cartographic models are created from topographic terrain models and presented through maps. In the cartographic model theory, the topographic terrain model is called the primary model and the cartographic model is called the secondary model. The model formed about the real world in the user's memory as a result of the interpretation of the cartographic model is called the tertiary model (or mental map)^40^. Thanks to the remote sensing platforms and applications that have developed with the advancement of technology, scientific studies have begun to result in an advanced way in larger areas. Geographical Information System (GIS) technology, which is one of the remote infrastructure platforms, has advanced in the emerging technology. Geographical Information System combines software, hardware, data and people on interactive maps; it is a complete system that allows viewing, viewing, querying and analysing geographic information and the data tables associated with them. This system provides faster and more reliable results in analysis by using more than one map layer^33^.

### Intuitionistic fuzzy sets

#### Definition 1

A fuzzy set $\stackrel{\sim }{A}$ in a universe of discourse X is defined as:

1 $$\stackrel{\sim }{A}= \left\{(x, {\mu }_{\stackrel{\sim }{A }}\left(x\right))|\forall x\in X\right\}$$

where ${\mu }_{\stackrel{\sim }{A }}\left(x\right):x\to [\mathrm{0,1}]$ as a membership value of $x\in X$ for a fuzzy set $\stackrel{\sim }{A}$^41^.

#### Definition 2

An intuitionistic fuzzy set (IFS)$\stackrel{\sim }{A}$ is defined as:

2 $$ (\mathrm{IFS})\stackrel{\sim }{A}= \left\{\left(x, {\mu }_{\stackrel{\sim }{A }}\left(x\right),{v}_{\stackrel{\sim }{A }}\left(x\right)\right)|\forall x\in X\right\}$$

where ${\mu }_{\stackrel{\sim }{A }}\left(x\right):x\to [\mathrm{0,1}]$ and ${v}_{\stackrel{\sim }{A }}\left(x\right):x\to [\mathrm{0,1}]$ under the condition $0\le {\mu }_{\stackrel{\sim }{A }}\left(x\right)+ {v}_{\stackrel{\sim }{A }}\left(x\right)\le 1$ for each $x\in X$ for a fuzzy set $(\mathrm{IFS})\stackrel{\sim }{A}$. ${\mu }_{\stackrel{\sim }{A }}\left(x\right)$ and ${v}_{\stackrel{\sim }{A }}\left(x\right)$ express the degree of membership and the degree of non-membership, respectively^42^.

#### Definition 3

The intuitionistic fuzzy hesitation (the non-determinacy) degree ${\pi }_{\stackrel{\sim }{A }}\left(x\right)$ is defined as:

3 $${\pi }_{\stackrel{\sim }{A }}\left(x\right)= 1- {\mu }_{\stackrel{\sim }{A }}\left(x\right)-{v}_{\stackrel{\sim }{A }}\left(x\right)$$

So, an intuitionistic fuzzy set can be shown as: $(\mathrm{IFS})\stackrel{\sim }{A}= {(\mu }_{\stackrel{\sim }{A }}\left(x\right), {v}_{\stackrel{\sim }{A }}\left(x\right) , {\pi }_{\stackrel{\sim }{A }}\left(x\right) )$ where ${\mu }_{\stackrel{\sim }{A }}\left(x\right):x\to [\mathrm{0,1}]$, ${v}_{\stackrel{\sim }{A }}\left(x\right):x\to [\mathrm{0,1}]$, $0\le {\mu }_{\stackrel{\sim }{A }}\left(x\right)+ {v}_{\stackrel{\sim }{A }}\left(x\right)\le 1$ and ${\pi }_{\stackrel{\sim }{A }}\left(x\right)=1- {\mu }_{\stackrel{\sim }{A }}\left(x\right)-{v}_{\stackrel{\sim }{A }}\left(x\right)$^42^.

### Intuitionistic fuzzy algebraic operations

Let consider two intuitionistic fuzzy sets $\stackrel{\sim }{A}={(\mu }_{\stackrel{\sim }{A }}\left(x\right), {v}_{\stackrel{\sim }{A }}\left(x\right) , {\pi }_{\stackrel{\sim }{A }}\left(x\right) )$ and $\stackrel{\sim }{B}={(\mu }_{\stackrel{\sim }{B }}\left(x\right), {v}_{\stackrel{\sim }{B }}\left(x\right) , {\pi }_{\stackrel{\sim }{B }}\left(x\right) )$ in the universe X. Then the algebraic operations used in this study are shown as follows^42^:

- $$\tilde{A} \otimes \tilde{B} = \left\{ {\left( {\mu_{{\widetilde{A }}} \left( x \right)v_{{\widetilde{B }}} \left( x \right)} \right), (v_{{\widetilde{A }}} \left( x \right) + v_{{\widetilde{B }}} \left( x \right) - v_{{\widetilde{A }}} \left( x \right)v_{{\widetilde{B }}} \left( x \right)), (1 - \mu_{{\widetilde{A }}} \left( x \right)\mu_{{\widetilde{B }}} \left( x \right) - \left( {v_{{\widetilde{A }}} \left( x \right) + v_{{\widetilde{B }}} \left( x \right) - v_{{\widetilde{A }}} \left( x \right)v_{{\widetilde{B }}} \left( x \right)} \right)} \right\} ,$$
- $${\stackrel{\sim }{A}}^{\lambda }=\left(({{\mu }_{\stackrel{\sim }{A }}\left(x\right)}^{\lambda }, 1-{\left(1-{ v}_{\stackrel{\sim }{A }}\left(x\right)\right)}^{\lambda }, {\left(1-{ v}_{\stackrel{\sim }{A }}\left(x\right)\right)}^{\lambda }-{{\mu }_{\stackrel{\sim }{A }}\left(x\right)}^{\lambda }\right), \lambda >0,$$
- $$\lambda \stackrel{\sim }{A}=\left(1-{\left(1-{\mu }_{\stackrel{\sim }{A }}\left(x\right)\right)}^{\lambda }, {{ v}_{\stackrel{\sim }{A }}\left(x\right)}^{\lambda }, {\left(1-{\mu }_{\stackrel{\sim }{A }}\left(x\right)\right)}^{\lambda }- {{ v}_{\stackrel{\sim }{A }}\left(x\right)}^{\lambda }\right), \lambda >0,$$

In this study, a normalised Euclidean distance^43^ of two intuitionistic fuzzy sets is used and intuitionistic fuzzy arithmetic weighted averaging operator is proposed to calculate an aggregated intuitionistic fuzzy decision matrix.

#### Definition 4

Let consider ${\stackrel{\sim }{A}}_{1}$, ${\stackrel{\sim }{A}}_{2}$, …., ${\stackrel{\sim }{A}}_{n}$ is an IFS where ${\stackrel{\sim }{A}}_{i}={(\mu }_{\stackrel{\sim }{{A}_{i}}}\left(x\right), {v}_{\stackrel{\sim }{{A}_{i}}}\left(x\right) , {\pi }_{\stackrel{\sim }{{A }_{i}}}\left(x\right) )$ and $i=\mathrm{1,2},\dots ,n$.The intuitionistic fuzzy arithmetic weighted averaging (IFWAA) operator is defined as:

4 $${I{FWAA}_{w}(\stackrel{\sim }{A}}_{1}, {\stackrel{\sim }{A}}_{2},\dots .,{\stackrel{\sim }{A}}_{n})= \sum_{i=1}^{n}{w}_{i}{\stackrel{\sim }{A}}_{i}=\left(1- \prod_{i=1}^{n}{\left(1- {\mu }_{\stackrel{\sim }{{A}_{i}}}\left(x\right)\right)}^{{w}_{i}}, \prod_{i=1}^{n}{{(v}_{\stackrel{\sim }{{A}_{i}}}\left(x\right))}^{{w}_{i}}, \prod_{i=1}^{n}{\left(1- {\mu }_{\stackrel{\sim }{{A}_{i}}}\left(x\right)\right)}^{{w}_{i}}- \prod_{i=1}^{n}{{(v}_{\stackrel{\sim }{{A}_{i}}}\left(x\right))}^{{w}_{i}}\right)$$

where ${w}_{i}$ is the weight of ${\stackrel{\sim }{A}}_{i}$.

### The motivation for using intuitionistic fuzzy sets

Atanassov^42^ presented an intuitionistic fuzzy set (A-IFS) characterized by a membership function and a non-membership function. The essential characteristic of an intuitionistic fuzzy set is to take into consideration not only membership degree but also non-membership degree of each element and it is one of extensions of fuzzy sets defined by Zadeh in 1965^44^. Gau and Buehrer^45^ stated that accuracy of fuzzy sets could be lost simply without considering non-membership degree. They also pointed out A-IFS named as a vague set can detect inconsistencies in assignments of elements keeping track of both membership and non-membership degrees. According to Wang and Li^46^, intuitionistic fuzzy sets are able to adapt user data without loss of decision information and the hesitancy degree can be used to handle uncertainty caused by the expert’s judgment ability. For this reason, a number of researchers as well as practitioners have preferred the A-IFS to apply into varying fields in the last two decades. For example, in order to cope with multi-criteria fuzzy decision-making problems, Lin et al.^47^ proposed an intuitionistic fuzzy set approach which evaluates each alternative with respect to degrees of satisfiability and non-satisfiability. Garg^48^ addressed an intuitionistic fuzzy decision making method investigating some existing aggregation operators for A-IFS and proposed a new aggregation operator which takes into account the hesitance between the membership functions to control the simultaneous effects on each other. Geng et al.^49^ presented a site selection framework for fish farms using interval-valued intuitionistic fuzzy sets and showed that they are able to handle uncertainties caused by inability to cover expert opinions, the independence assumptions and the diversity of information. Schitea et al.^50^ proposed an intuitionistic fuzzy set based multi-criteria decision making approach in order to investigate location in Romania for hydrogen mobility roll-up stations. Hence, in this study, an intuitionistic fuzzy set approach is used and to the best of authors’ knowledge, this is one of the first studies apply the A-IFS to a site selection problem for the solar energy power plant installation. The proposed approach is also employed to solve a real-world problem instance from Erzurum province in Turkey.

### Objectives of the study

The field of geographic information systems has become extremely useful in understanding the bigger picture of lands. GIS also has been used to map the solar potential of different areas in the world. In addition, the majority of the previous works on site selection for solar energy sources are generally used either type-1 fuzzy sets or combination with other multi-criteria decision making methods. Generally, these works differ based on criteria selected and geographical constraints. In this study, we introduce novel multi-stage approach based on intuitionistic fuzzy sets for solar energy power plant potential site selection along with mapping Erzurum province in terms of solar sources using GIS. The main objectives of this study are to:

1. Make a solar energy power plant potential site selection map using a GIS program in the provincial scale by taking into account ecological risks and ecological criteria in a way to ensure the least destruction on the natural balance of Erzurum province.
2. Examine 20districts of Erzurum as alternatives using intuitionistic fuzzy sets along with 10 criteria identified and their relative importance determined.
3. Check consistency of results obtained by two methods comparing them to each other.

The rest of the paper is structured as follows. Section “Background” describes the real-world problem including its description, the criteria selected and alternatives determined to evaluate. In “Problem description”, the overall approach is introduced. Section “Methodology” explains the methodology for GIS and intuitionistic fuzzy approaches. And then, the results of the study are given with a comparison of two approaches proposed in “Preliminary experiments and results”. Finally, “Experimental results” concludes the study and presents some potential future research directions.

### Problem description

**Figure 2:**
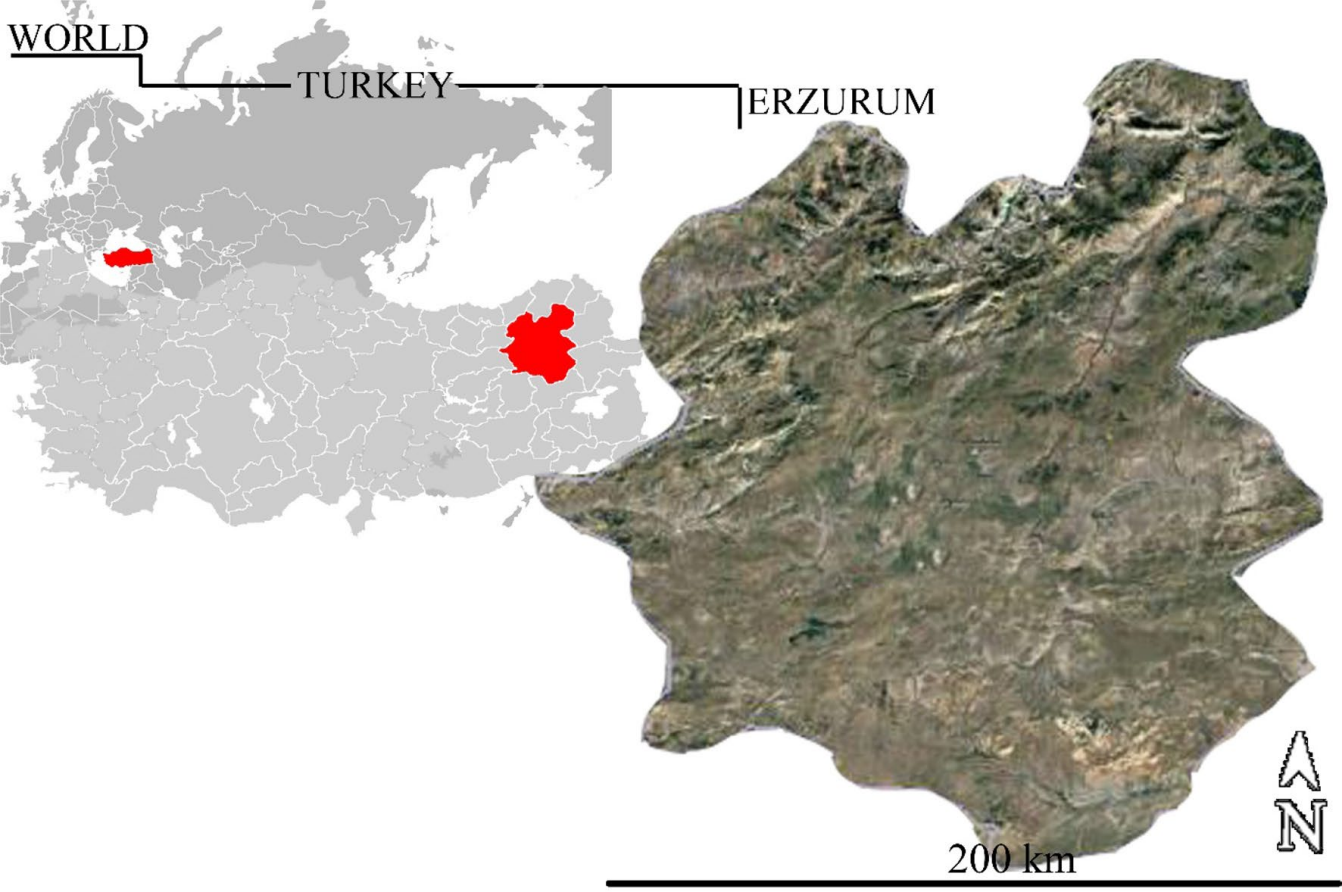
Location map of Erzurum as defined as the study area.

The material of this study area constitutes the Erzurum province. The province of Erzurum is between the latitudes of 40° 14′ 15′ and 42° 33′ 35′ east and 40° 54′ 57′ and 39° 06′ 10′ north latitudes and has a Erzurum areas approximately 25.355 km^2^ as seen in Fig. 2.

Erzurum province is between approximately 800 m to 3800 m above sea level. It consists of 63.7% mountains (Rize Mountains, North Anatolian Mountains, Karasu-Aras Mountains), 4% plains (Erzurum Plain, Pasinler Plain, Hınıs Plain), 12.2% plateaus in terms of landforms. 20% of its lands also create plateaus. Approximately 26.5% (656,782.6 ha) slope of the province constitutes areas with an embroidered agricultural limit and below 12%.

### Criteria for decision making

**Figure 3:**
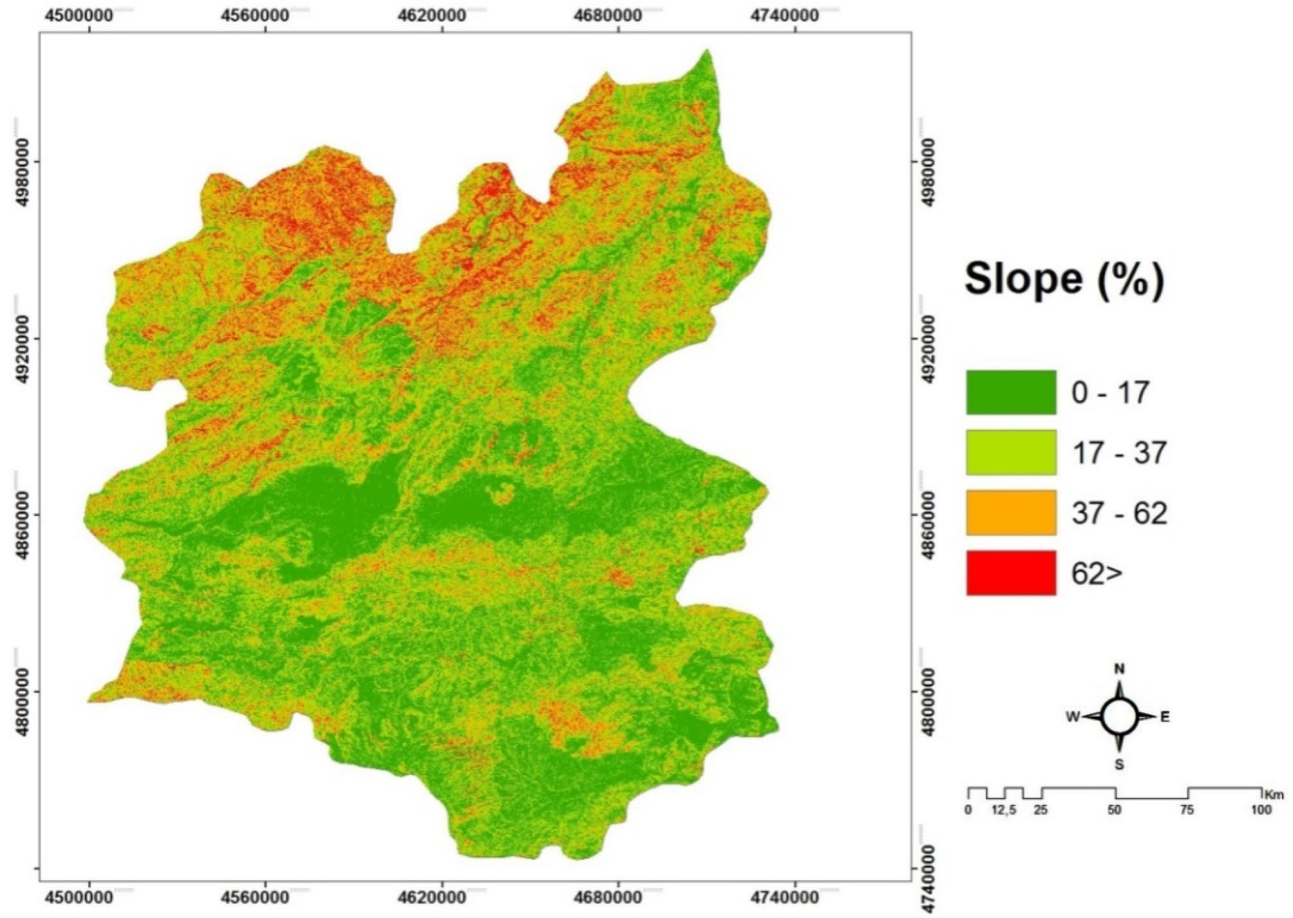
The slope map of Erzurum/Turkey.

We identified 10 criteria with respect to extant literature and expert opinion for the solar energy power plant site selection problem of Erzurum province. These criteria are considered in this study as summarised in the following:

*(C1) Slope* is defined as a measurement of steepness or inclination to the horizontal plane. As the temperature rises every 200 m, it decreases by 1 °C on average; the effect of the slope is on permanent snow in the high parts of the mountains, difference of temperatures in different latitudes in the same longitude and the amount of rainfall^20^. The slope map of Erzurum is shown in Fig. 3.

**Figure 4:**
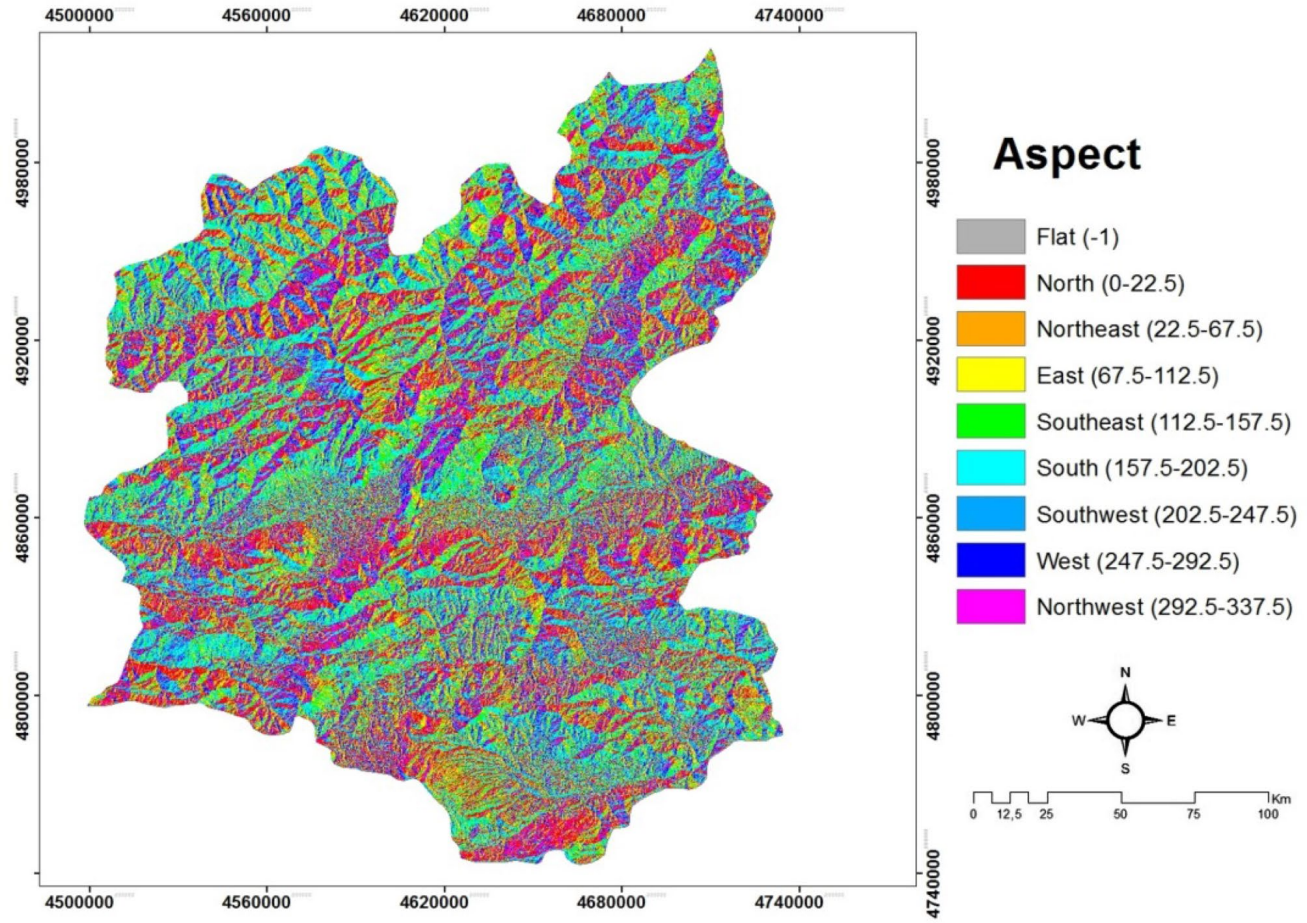
The aspect map of Erzurum/Turkey.

*(C2) Aspect* can be explained as the direction of sun exposure of the mountains or the view of the mountains in a region. Shortly, it is the position of a place against the sun, which in turn affects the time of sunbathing. Sunbathing time in a place also affects the air temperature in residential and business places in that place. In addition, it has effects on the amount of fuel in a settlement for heating to sustain the desirable level of temperature. As such, the relationship between inspection conditions and air pollution emerges spontaneously^20^. The aspect map of Erzurum province is indicated in Fig. 4.

**Figure 5:**
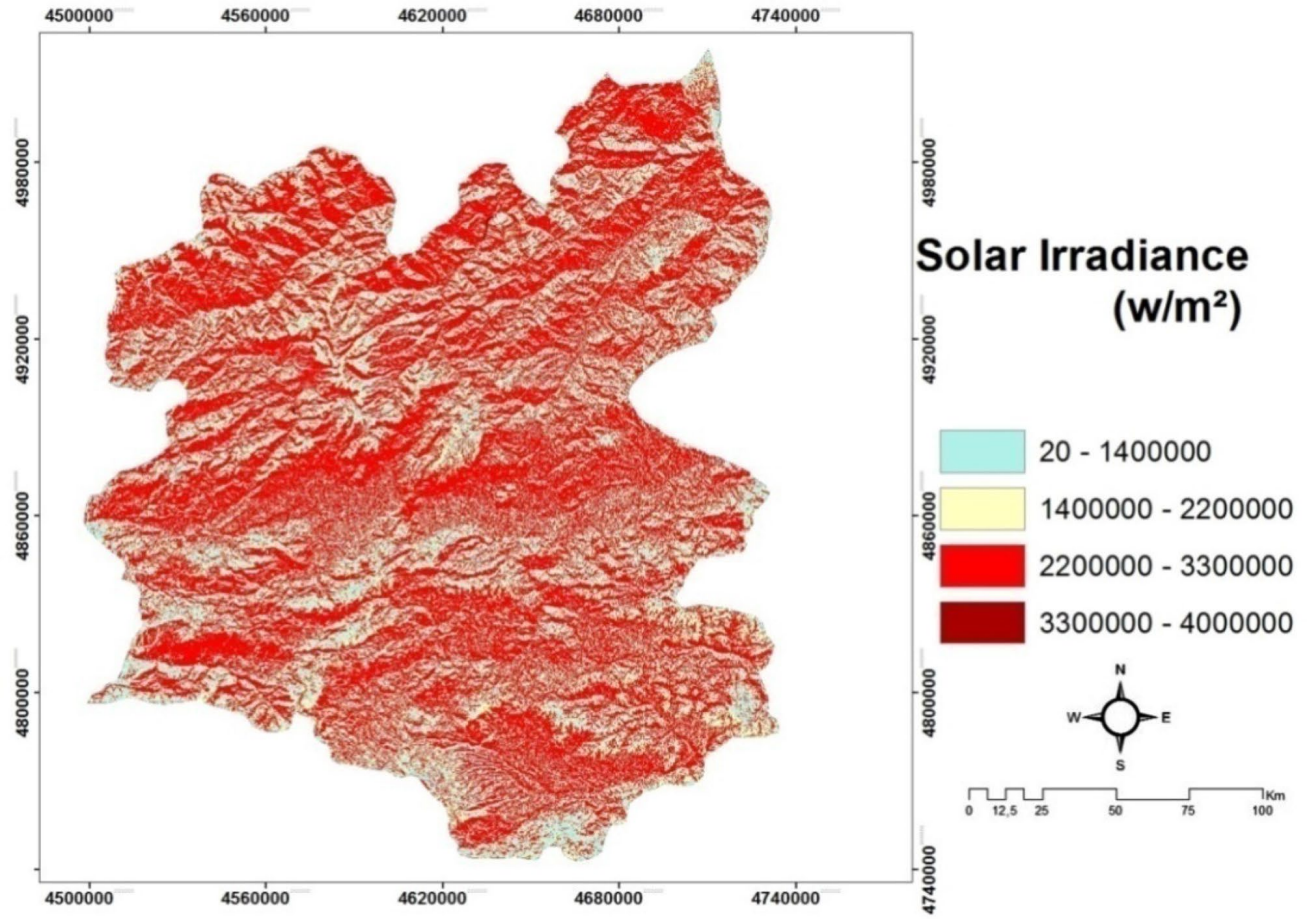
The map for solar irradiance in Erzurum.

*(C3) Solar irradiation *is one of the most important aspects for solar energy. It is associated with the duration of sunbathing time. The longer the sunbathing time is related to the higher the temperature. Despite the fact that the polar are experienced for 6 months day and night, the temperature is still low because the sun's rays are tilted to the ground^20^. For Erzurum, Fig. 5 demonstrates its solar irradiation map.

**Figure 6:**
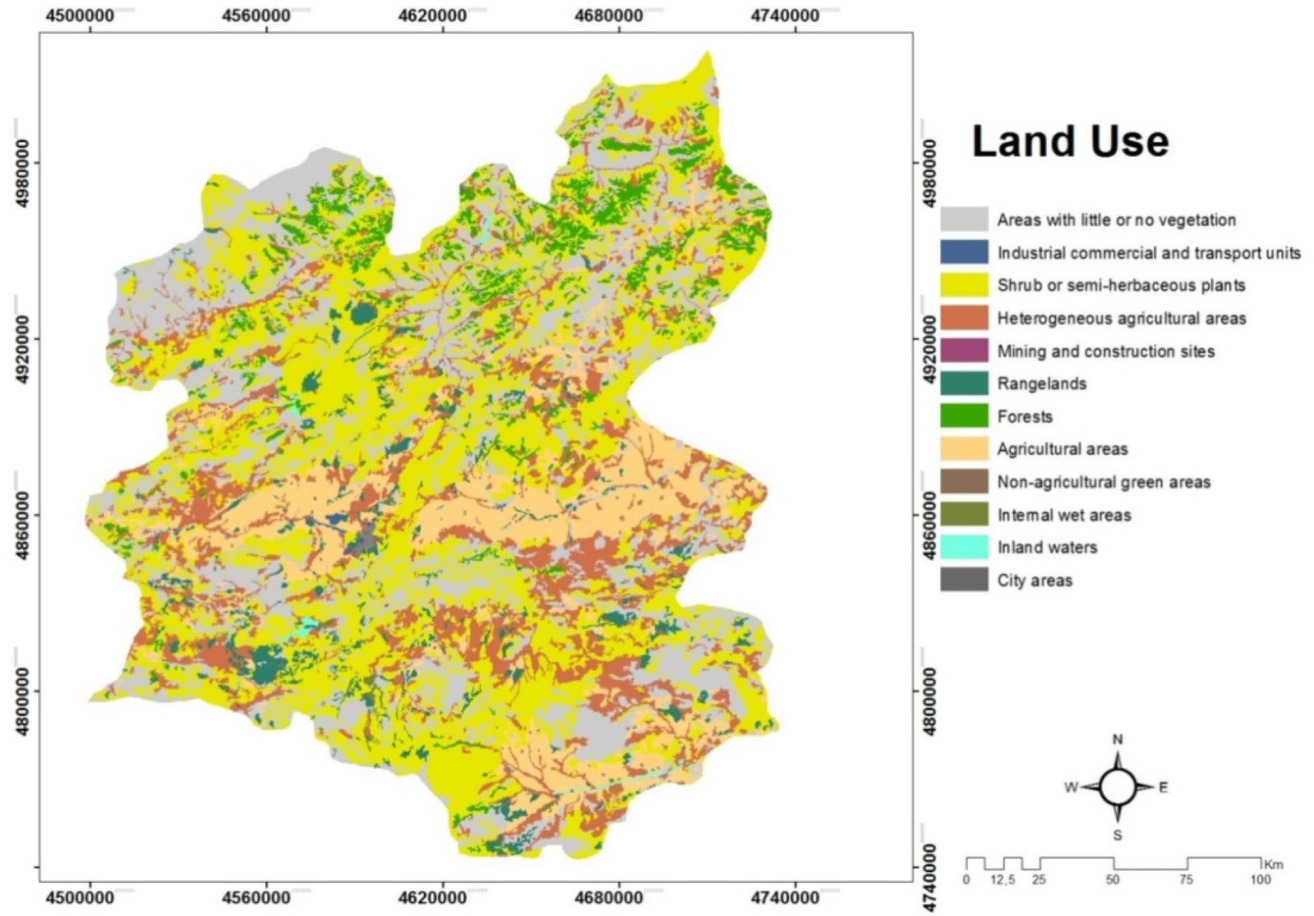
The map of land use Erzurum province/Turkey.

*(C4) Land use* is about soil structure of land. Being stony (rocky) or soil defines the quality of the land. For example, when the rocky parts are too much, the constructions have to be used along with concrete feet and this increases the cost by 30%. The sun-wet soil heats up quickly, cools quickly, and gets very hot, very cold. This situation affects the surface temperature of the land. The rapidly warming surface radiates heat faster and allows the solar panels to warm up from behind. Heat and energy loss is high in the solar panel, which is heated from the back. In order to avoid high energy loss, the temperature of the land surface area should be low. In addition, for solar power plants, it is essential to have a southern facade in order to make maximum use of the solar potential^20^. Figure 6 shows the map of Erzurum province in terms of land use.

**Figure 7:**
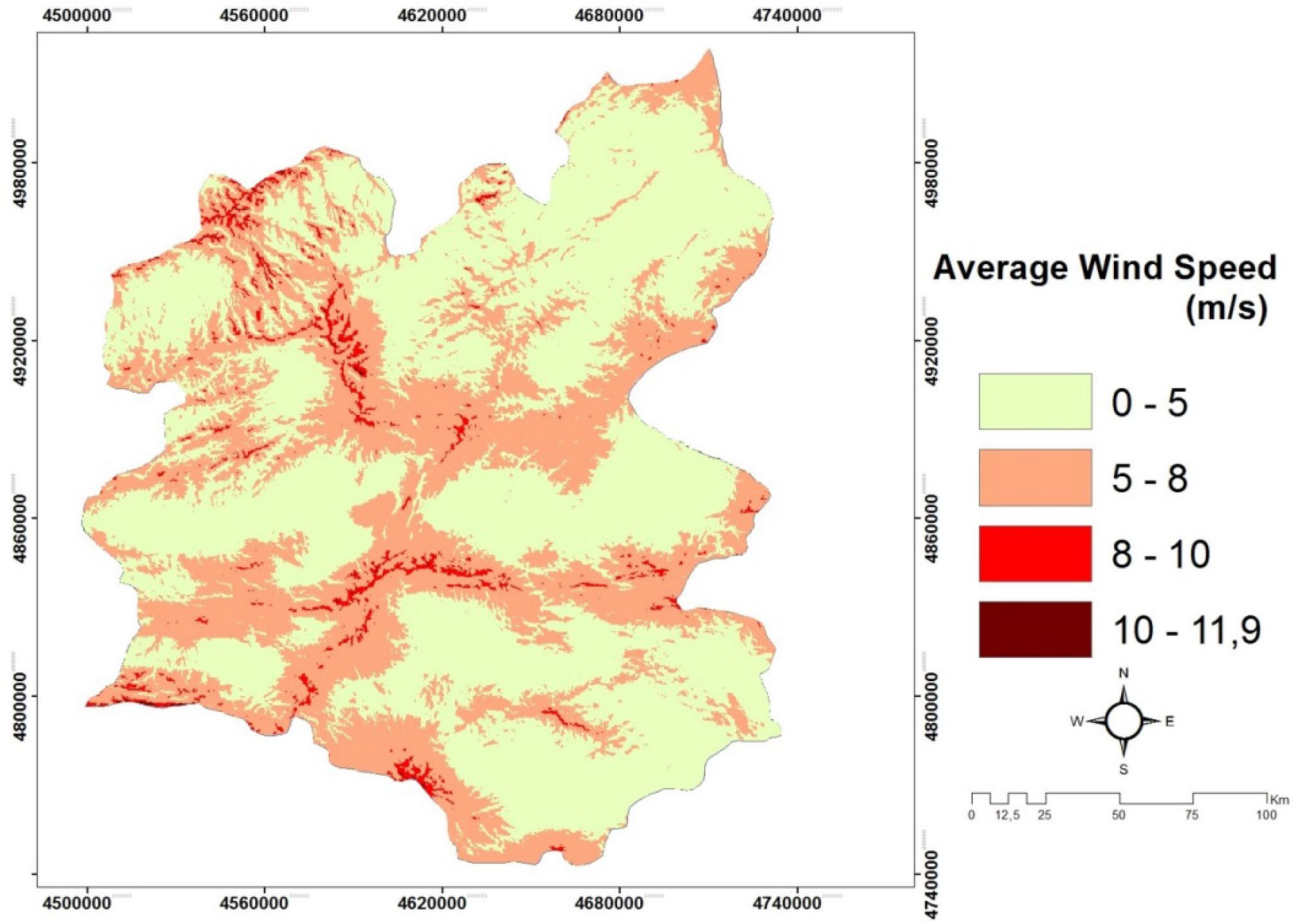
The average wind speed of Erzurum/Turkey.

*(C5) Wind speed* is one of the most significant aspects to reduce the temperature of PVs for solar power plants. Determining the direction of the wind is also essential to adjust solar panels with a right angle^33,51,52^. The average wind speed map of Erzurum is demonstrated in Fig. 7.

**Figure 8:**
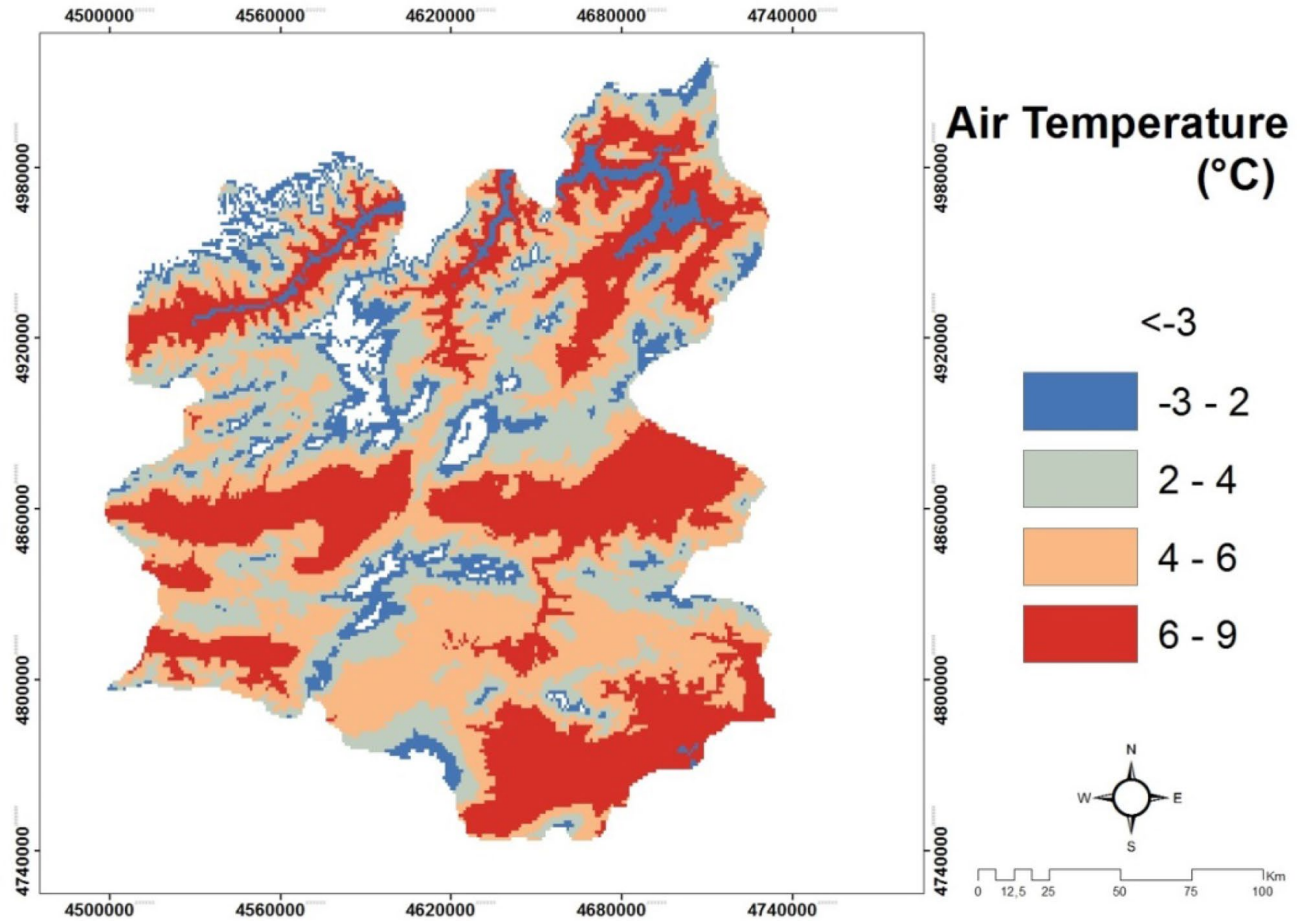
The temperature map of Erzurum/Turkey.

*(C6) Air Temperature *has magnificent influence on human and all other livings as well as for inanimate beings and various events. Temperature is also essential in economic and social activities and natural phenomenon such as the dissolution of rocks. The temperature is not evenly distributed throughout the world. So, some places are desirable in terms of temperature they have for human beings. This is also valid for solar power plant installation^20,43^. In Fig. 8, the temperature map of Erzurum is demonstrated.

**Figure 9:**
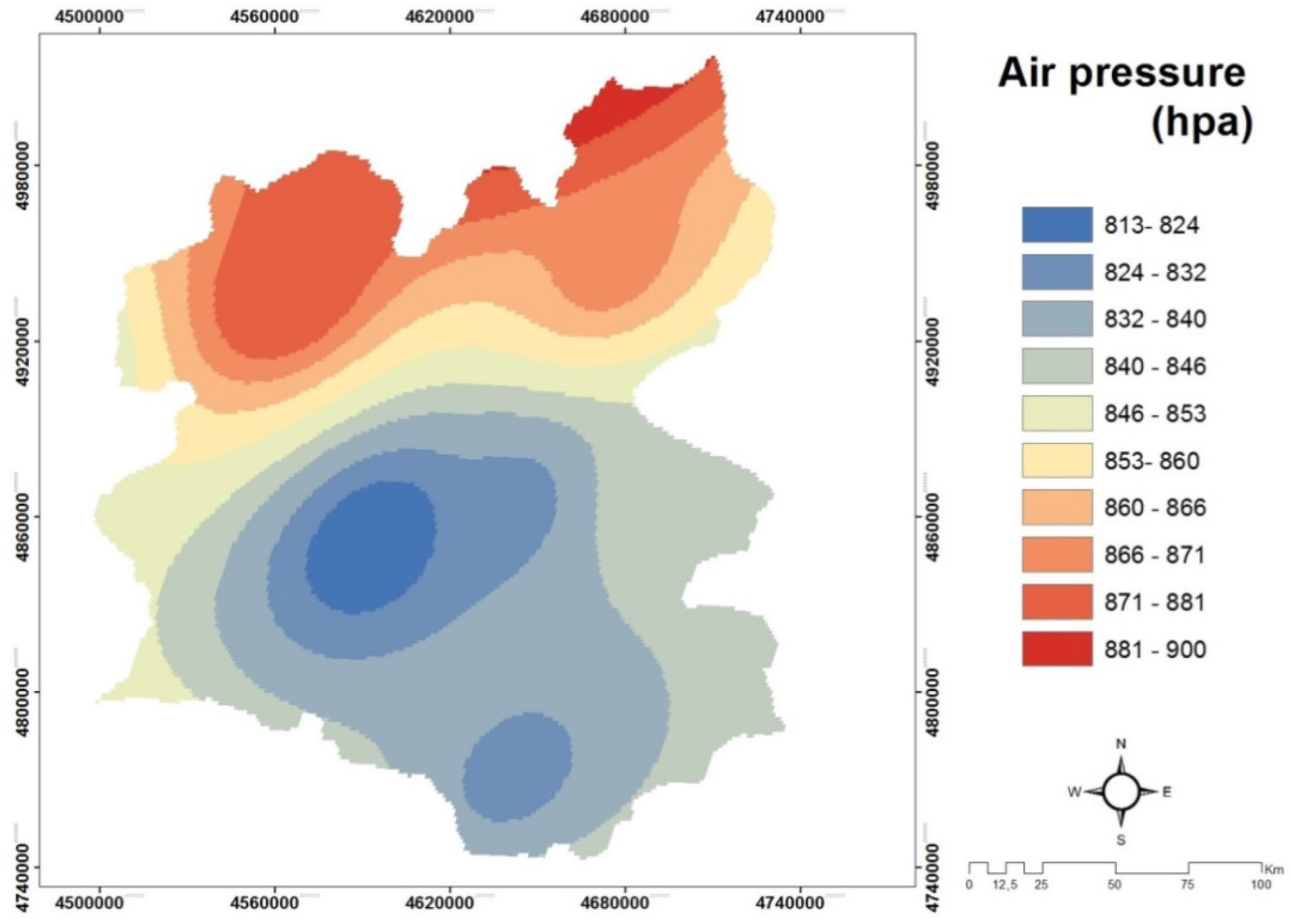
The air pressure map of Erzurum^20,43^.

*(C7) Air Pressure *Air pressure (in Fig. 9) is the weight of air above the surface and the force per unit of area exerted on the Earth's surface. The force exerted by an air mass, the molecules that make up it and their size; movement and number are found in the air. These factors are important because they determine the temperature and density of the air and therefore its pressure. That is, the number of air molecules on a surface determines the air pressure. The higher the molecular number, the more pressure it exerts on a surface and the higher the total atmospheric pressure. Conversely, if the number of molecules decreases, so does the air pressure. As the air density increases, the pressure increases. Air movements, water vapour and dust in the air increase the pressure. Air pressure does not show a uniform distribution according to latitude^51^.

**Figure 10:**
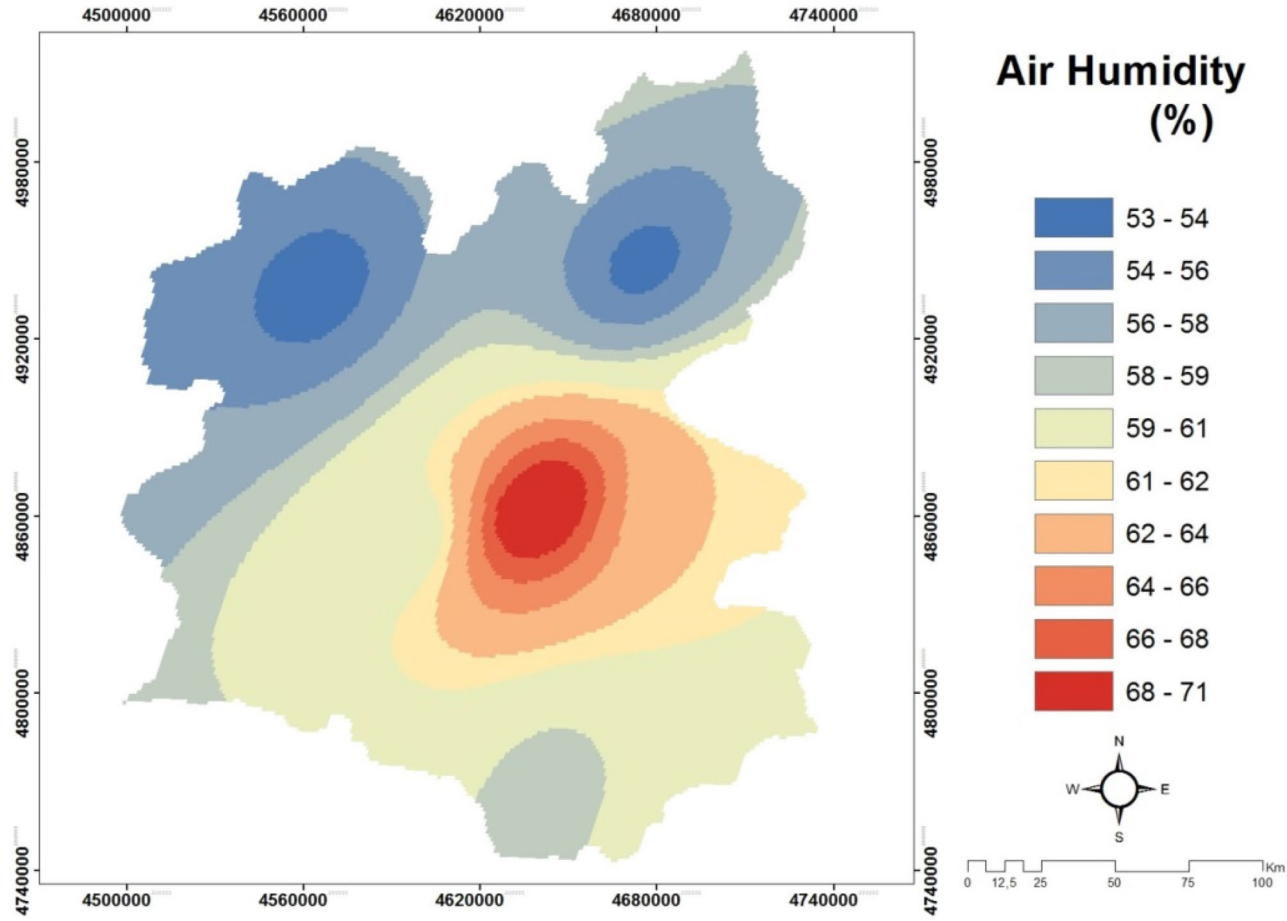
The air humidity map of Erzurum^20,50^.

*(C8) Air Humidity* The water vapor carried by the air is called air humidity (Fig. 10). The amount of moisture that the air can carry, namely the amount of water vapor, changes with temperature and pressure. Increasing temperature increases the amount of moisture that can be transported. When relative humidity reaches 100%, it means that the air has reached the maximum amount of moisture it can carry, that is, it is saturated. There is no chance of getting any more water inside. If there is still water in the environment trying to join the air, the vapor will condense and become liquid. Rain formation happens in this way. The higher the relative humidity, the more likely it is to rain^51^.

**Figure 11:**
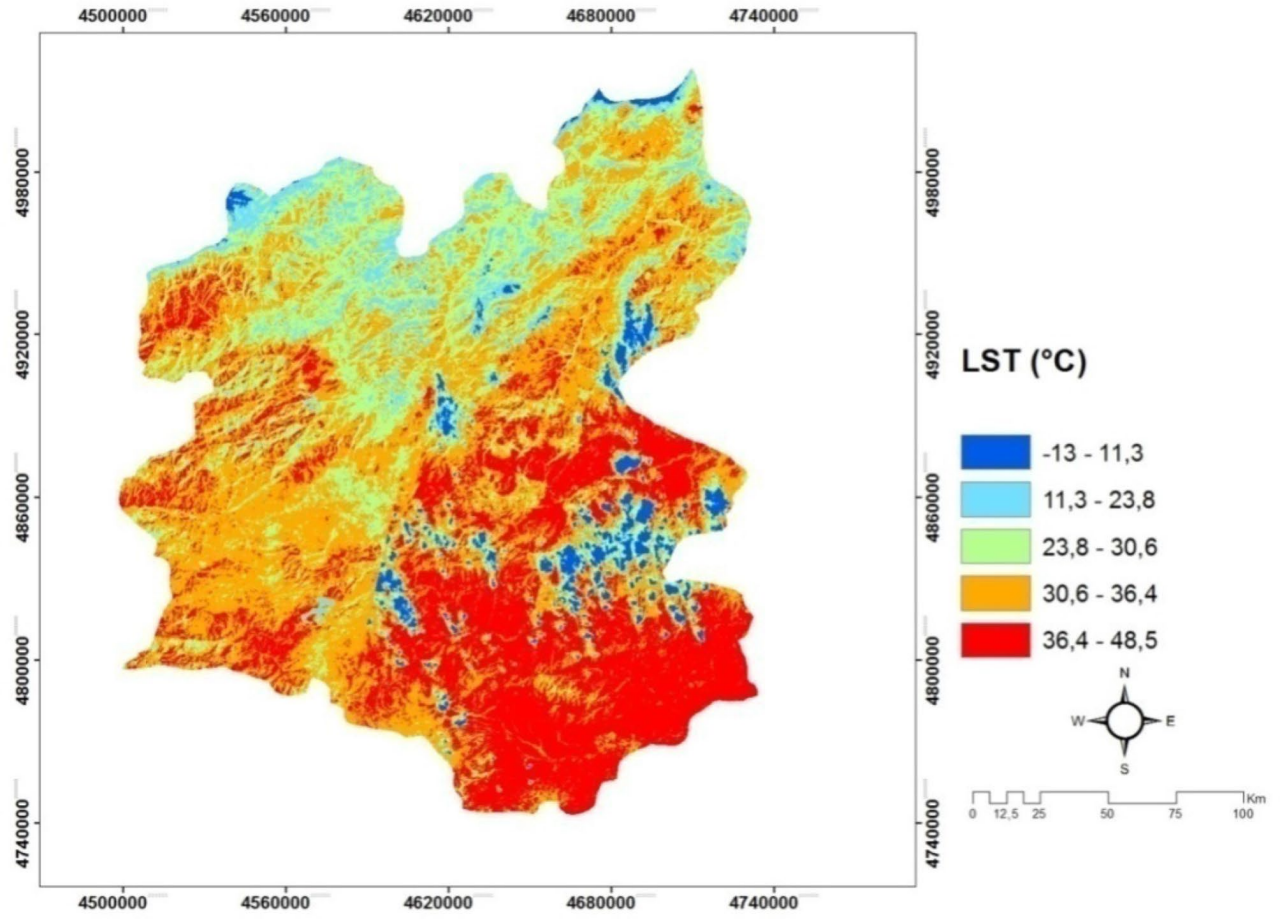
The Land surface temperature (LST) map of Erzurum^20,43^.

*(C9) Land surface temperature* Ground Surface Temperature (GST) is a very important component that manages the world energy balance. Numerous methods have been developed to obtain ground surface temperature with remotely sensed images with thermal band. In literature, there are various methods; split-window method, temperature/emissivity separation method, single-window (mono-window) algorithm, single channel (single channel) method. In this study, we used the surface thermal temperature in the selection of the solar power plants as seen Fig. 11. In general, thermal bands of satellite images are used to collect data for dense urban areas, determination of city heat islands, and determination of the effects of land cover and use changes on the regional climate, analysis of climate changes, analysis of global warming effects, ecological studies, human health studies, effective energy use studies, etc.

**Figure 12:**
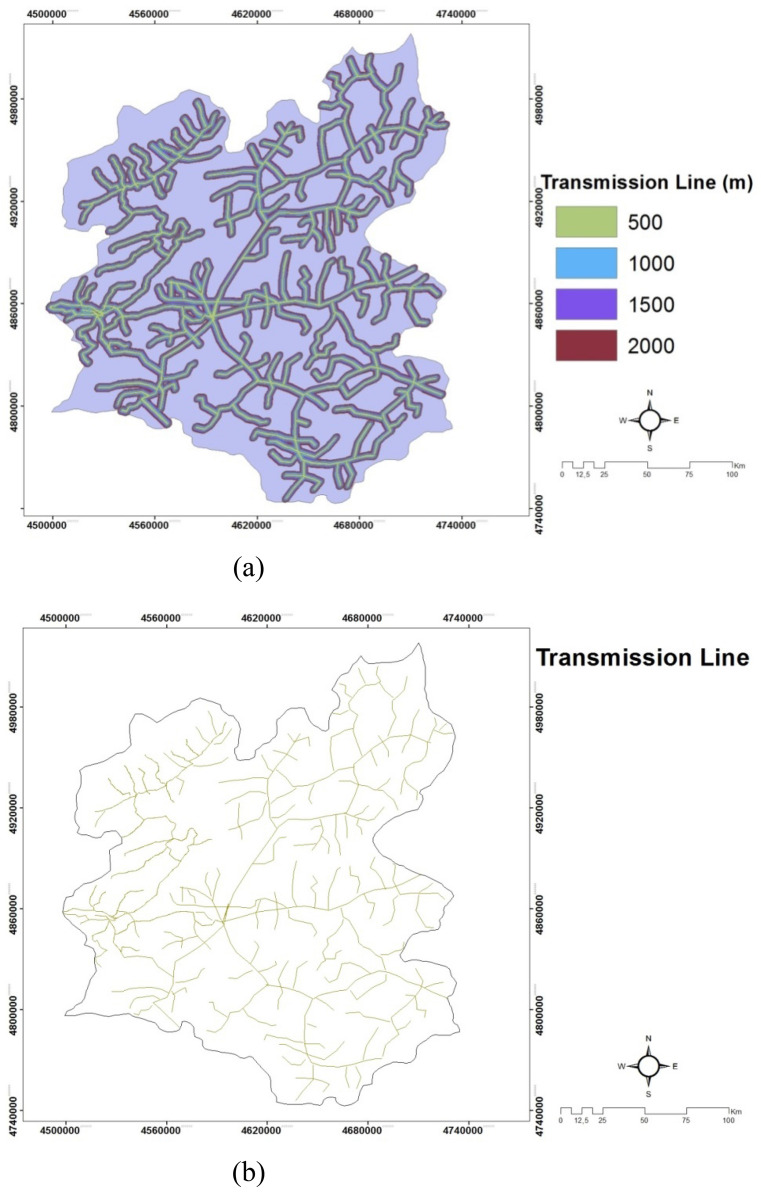
(**a**) The transmission line that we obtained with CSB method map of Erzurum. (**b**) The transmission mains of power line map of Erzurum^20,52^.

*(C10) Transmission line* is defined as that the lines ensure the transmission of controlled and planned electrical energy from the power plants to the distribution lines. It is also desirable that having electricity generation facilities and transformer stations near the electricity consumption zones. It is very important to construct the power line safely and transmit the electricity with minimum losses. Issues such as cost, route of the transmission line, land condition, security element of the line are examined in laying the electrical lines. Another important parameter in the installation locations of solar power plants is the distance of solar power plants to electricity transmission lines. If the solar power plant to be installed is far from the transmission lines, it increases the energy installation cost due to the cable costs. In order to avoid this cost increase, places close to the transmission lines of the solar power plant to be installed are selected. For Erzurum, the maps for transmission lines are indicated in Fig. 12a and b. The raster datas of Turkey Electricity Distribution companies have been digitized in ArcGIS 10.2 software package. These digitized data were analysed at a distance in the BUffer Analysis program.

### Alternative options

In this study, it is aimed to solve the problem of Solar Power Plant Location Selection at the provincial scale, taking into account ecological risks, ecological criteria and its proximity to electrical transmission line in order to minimize the damage to the natural balance of Erzurum province/Turkey. In this study, we have 20 districts as alternatives to consider placing a solar power plant installation based on the real-world data of Erzurum province, Turkey. The names of districts can be listed as Askale (A1), Aziziye (A2), Cat (A3), Hinis (A4), Horasan(A5), Ispir (A6), Karacoban (A7), Karayazi (A8), Koprukoy (A9), Narman (A10), Oltu (A11), Olur (A12), Palandoken (A13), Pasinler (A14), Pazaryolu (A15), Senkaya (A16), Tekman (A17), Tortum (A18), Uzundere (A19) and Yakutiye (A20).

## Methodology

This section describes the GIS methodology used and fuzzy approach proposed in this study.

### Methodology for GIS

**Figure 13:**
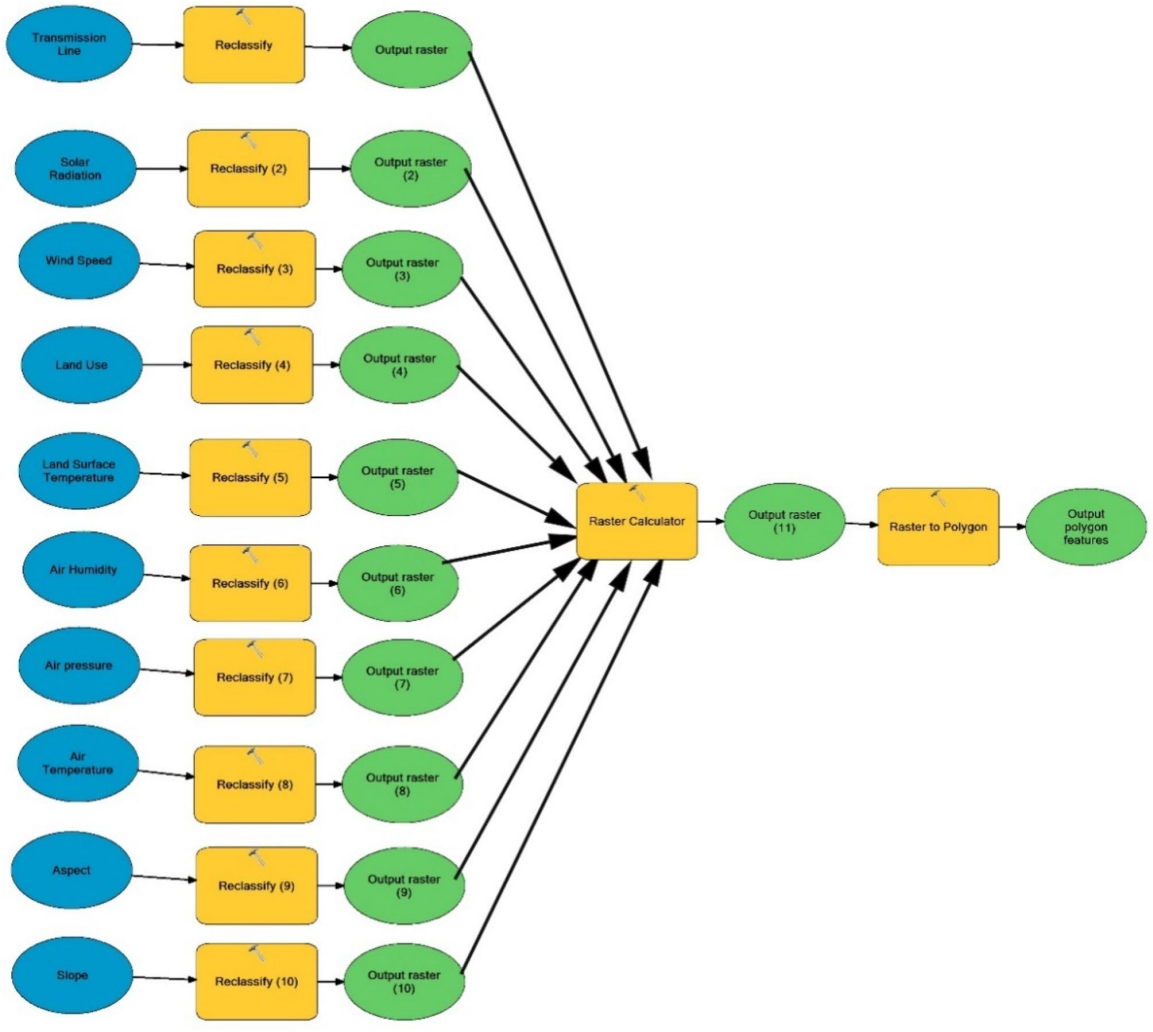
Model created for study result maps.

In the study, raster data sets and digital data are evaluated after a model is created in Arc-GIS 10.2 software. In this model created, data order and analysis are shown in Fig. 13. Before the model is generated, raster transformations are performed to analyze all data sets with (C1) Slope, (C2) Aspect, (C3) Solar irradiation, (C4) Land use, (C5) Wind Speed, (C6) Air Temperature, (C7) Air Pressure, (C8) Air Humidity, (C9) Land Surface Temperature (LST), (C10) Transmission Line, respectively. Classification is made for each layer after raster transformations. As it can be seen in the model created for classification, the weight of each layer is determined by giving a score of 1–4 for the components of each layer according to the degree of weight, and the result map is created by classifying after the layers were combined (seen in Table 1).

**Table 1:** Solar power plant scoring values created by experts for each layer.

Evaluation factor	Selected sub-units	Relevance number
Slope	Flat	4
	Slightly sloping	4
	Sloping	3
	Very sloping	1
Aspect	South	4
	South West	2
	South East	2
	North	1
	North East	1
	North West	1
	West	1
	East	1
	Flat	1
Solar irradiation	Irradiation excess > 42,000,000 W/m^2^	4
	Irradiation too 42,000,000–3,000,000 W/m^2^	3
	Irradiation normal 3,000,000–100,000 W/m^2^	2
	Irradiation low < 100,000 W/m^2^	1
Land use	City structure	1
	Industrial commercial and transports	1
	Mine and construction areas	1
	Non-agricultural artificial green areas	2
	Fields suitable for agriculture	1
	Continuous products	1
	Pasture area	3
	Heterogeneous agricultural areas	1
	Forests	1
	Shrubbery areas	2
	Areas without vegetation	3
	Interior wet areas	1
	Wet areas near the shore	1
	Inland waters	1
	Sea waters	1
Wind speed	Hard wind (10–12)	3
	Windy (8–10)	4
	Wind light (5–8)	4
	No wind or low (0–5)	1
Air temperature	Hot	2
	A bit hot	4
	Normal	4
	Cool	2
Air pressure	High	4
	Normal	3
	Low	2
	Very low	1
Air humidity	High	1
	Normal	2
	Low	3
	Very low	4
Land surface Temperature (LST)	So hot	1
	Hot	2
	Normal	3
	Cool	4
Transmission line	0–500 m	4
	500–1000 m	3
	1000–1500 m	2
	1500–2000 m	1
	2000 m >	1

#### Creating slope, aspect and solar irradiation maps

The slope and aspect maps of the study site are digitized in the Arc GIS 10.2 package program as a result of obtaining DEM maps with a spatial resolution of 30 m of ASTER satellite. Aspect and Slope and Solar Radiation maps are created in the Arc GIS 10.2 package program using the 'Spatial Analyst Tools' module. These maps generated are shown in Figs. 3, 4 and 5, respectively.

The slope maps (Fig. 3) were made using dwm datas of Landsat TM 8 satellite. These data were evaluated in the Arc GIS 10.2 package program, in the Spatial Analysis module, in the slope tool.

The solar irradiation (Fig. 5) from the sun undergoes changes first in the atmosphere and then in topography. It reaches the earth directly, diffused and reflected. The sum of these three components makes up global solar irradiation. In their analysis, solar radiation tools do not take into account the amount of solar irradiation reflected. It represents global solar irradiation only as the sum of direct and scattered solar irradiation. Solar irradiation analysis tools calculate solar charts by following the steps below.

- Calculation of an upward-facing hemisphere field of view based on topography.
- Overlay of the field of view placed directly on the sun chart to predict direct irradiation.
- Overlaying the field of view on a scattered sky map to estimate scattered irradiation.
- Repeating the process for each location of interest to generate a sunbathing map

In this study, NASA Shuttle Radar Topographic Mission (SRTM) 90 m × 90 m digital elevation model (DEM) of Erzurum province was used to model the direct solar irradiation map.

#### Creating a land use map

Existing land use maps have been utilized from the CORINE land cover system built on a 3-level hierarchical basis. Each level corresponds to an individual scale. Level III collects 1/100,000 scale information in 44 categories. Level II corresponds to the scales 1/500,000 and Level I 1/1,000,000. In this context, it was adapted to the current study area in 15 categories by reducing it to level 2 which was obtained as level 3. The land use map for Erzurum is demonstrated in Fig. 6.

#### Creating wind speed and air temperature maps

The wind map of the study area is obtained using Global wind Atlas (https://globalwindatlas.info/) data unit. The data obtained in numerical format is used by classification in Arc GIS package program. At the same time, the air temperature data sets are gathered from the resources of the Global Solar Atlas website (https://globalsolaratlas.info/map) and the classification is made. Figures 7 and 8 show the average wind speed map and the air temperature map for Erzurum province.

#### Creating air pressure and air humidity maps

**Figure 14:**
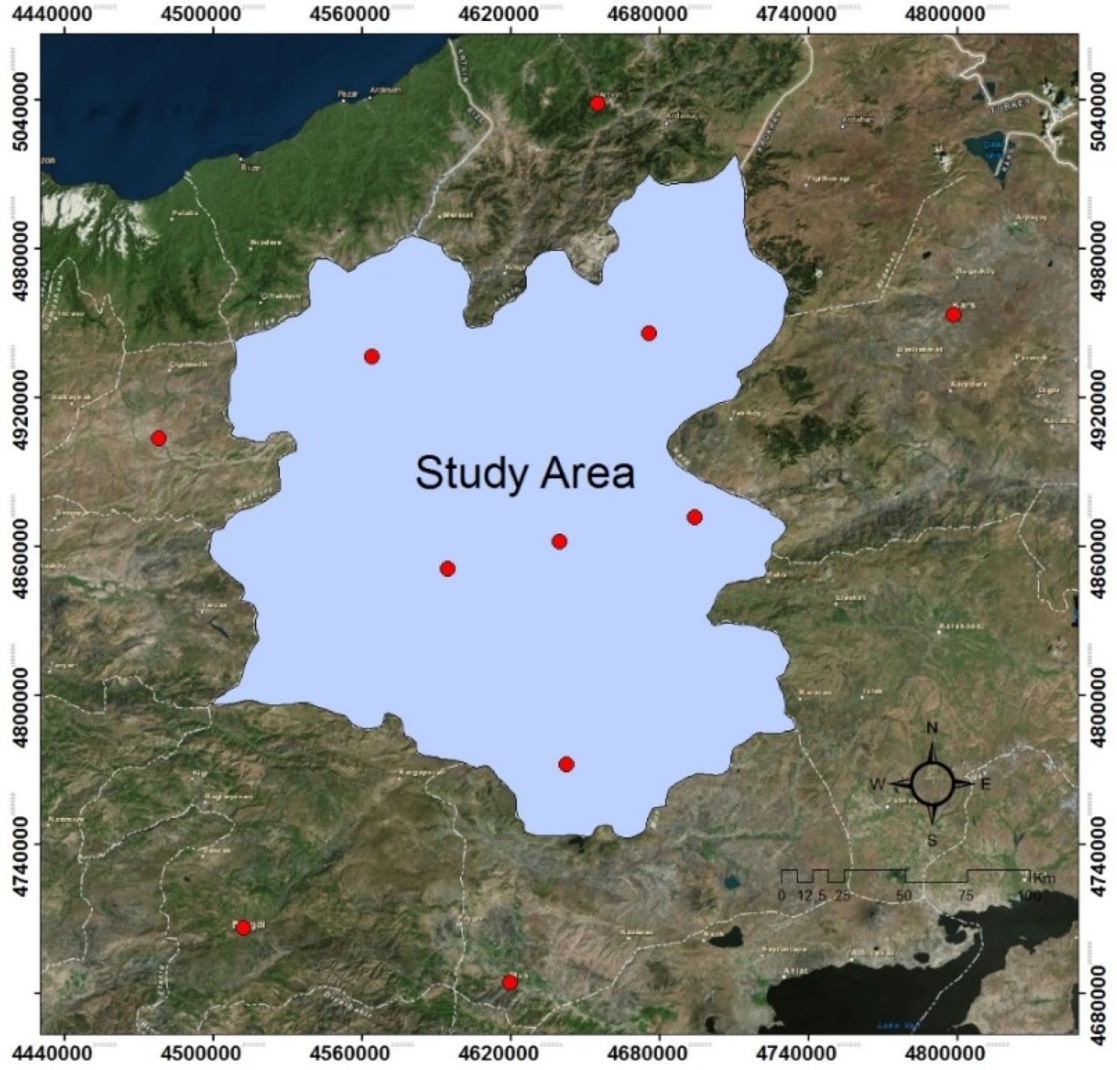
Sampling points used to create pressure and humidity maps.

While creating air pressure and humidity maps, 6 points are determined within the boundaries of the working area and 5 points outside the working boundary (Fig. 14). The annual average humidity and air pressure data of the determined points are obtained from the General Directorate of State Meteorological Affairs and their spatial distributions are created and mapped using the Kriging method in the Geostatistical Analyst module in the ArcGIS 10.2 package program.

#### Creating land surface temperature (LST) maps

**Figure 15:**
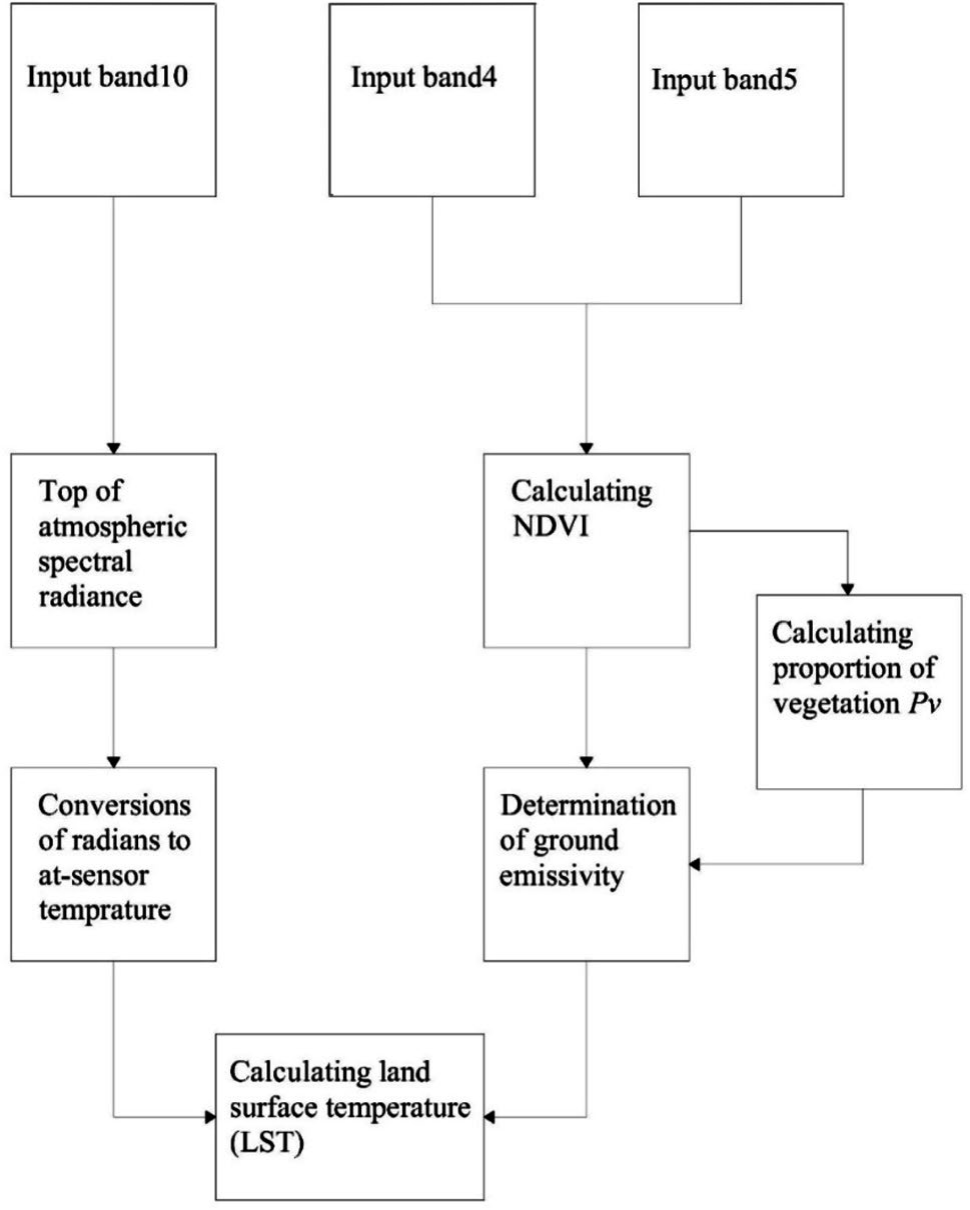
The schematic algorithm of Arc GIS package program.

LST maps of Land sat TM 8 satellite 30 m spatial resolution band 4, band 5 and band 10 data are used (USGS, 2016). Based on these data, procedures for previous studies are applied for the spatial distribution of surface temperature. The procedure applied is schematic as follows and shaped in the ArcGIS package program (as seen in Fig. 15).

The following equations are used in order to derive LST maps from band combinations of Landsat TM 8 satellite of the study area. In the first stage, the atmospheric radiation values are calculates as follows:

5 $$L\lambda =Band10 X 0.003342+0.1$$

where $L\lambda$ represents Spectral brightness in sensor opening (W/(m^2^ srμm)).In the second stage, the following equation is used to convert the atmospheric radiation value to sensor temperature (TB).

6 $${T}_{B}=\frac{1321.08}{\mathrm{Ln}((774.89/(\mathrm{ L\lambda }))+1)}-273.15$$

In the third stage, the following equation is used to obtain NDVI maps. Normalized plant density index (NDVI) maps are important since they show differences in temperature fluctuations of the natural surface compared to plant densities.

7 $$NDVI=\frac{Floot\left(Band5-Band4\right)}{Floot(Band5+Band4)}$$

In the fourth stage, the calculation of the vegetation rate is done using the equation shown in the followings:

8 $${P}_{v}=0.004X\left(NDVI\right)+0.986$$

where ${P}_{v}$ indicates the calculation of vegetation rate. Finally, the surface temperature maps (LST) is generated with a minimum margin of error using the following equation:

9 $$LST=\frac{{T}_{B}}{(1+\left(0.00115\mathrm{x}\frac{\mathrm{TB}}{1.4388}\right)Ln\left({P}_{v}\right))}$$

### Creating existing energy lines maps

Raster format of energy lines are 1/25,000 scale map of the power lines are obtained from the Turkey Electricity Distribution Company. The maps obtained are adapted according to WGS-84 projection zone 37 and zone 38, and the 14 maps obtained as a result of these adaptations are combined and electricity distribution line maps are created within the boundaries of the working area. These created maps are digitized in ArcGIS 10.2 package program. Digitized electricity distribution lines maps are shown in Fig. 12a and b.

### Methodology for intuitionistic fuzzy sets

In this study, we introduce an intuitionistic fuzzy approach for selecting desirable area for solar power plants in Erzurum, Turkey. In this sub-section, we explain the details of our approach starting with the intuitionistic fuzzy membership functions then the intuitionistic fuzzy approach proposed for ranking alternatives.

#### Fuzzy membership functions

In this study, we consulted and did a survey with a decision-maker and 10 criteria are selected for the overall evaluation process of 20 districts of Erzurum as the alternatives for the solar power plant installation problem.

Firstly, importance of the each criterion is rated using the linguistic terms “Very important (VI)”, “Important (I)”, “Medium (M)”, “Unimportant (U)”, “Very unimportant (VU)” as seen Table 2. Table 2 demonstrates the corresponding intuitionistic fuzzy sets, specified using a real value between 0 and 1 and Table 3 shows linguistic decisions of the decision-maker for the importance of criteria.

**Table 2:** Linguistic variables for evaluating each criterion^53^.

Linguistic variables	IFNs ($\mu , v,\pi )$
Very important (VI)	(1, 0, 0)
Important (I)	(0.75, 0.20, 0.05)
Medium (M)	(0.50, 0.40, 0.10)
Unimportant (U)	(0.25, 0.60, 0.15)
Very unimportant (VU)	(0.10, 0.80, 0.10)

**Table 3:** Importance of criteria according to the decision-maker.

	C1	C2	C3	C4	C5	C6	C7	C8	C9	C10
Decision maker	I	I	VI	VI	VI	VI	I	I	I	I

After deciding the importance of each criterion, the decision-maker provides a performance evaluation of district in terms of 10 criteria using the linguistic terms “Extreme high (EH)”, “Very high (VH)”, “High (H)”, “Medium high (MH)”, “Medium (M)”, “Medium low (ML)”, “Low(L)”, “Very low (VL)”, “Extreme low (EL)” and Table 4 shows the associated intuitionistic fuzzy sets for each term. Table 5 denotes linguistic decisions of the decision-maker for the performance of alternatives.

**Table 4:** Linguistic variables for evaluating each alternative^53^.

Linguistic variables	IFNs ($\mu , v,\pi )$
Extreme high (EH)	(0.95, 0.05, 0.00)
Very high (VH)	(0.85, 0.10, 0.05)
High (H)	(0.75, 0.15, 0.10)
Medium high (MH)	(0.65, 0.25, 0.10)
Medium (M)	(0.50, 0.40, 0.10)
Medium low (ML)	(0.35, 0.55, 0.10)
Low (L)	(0.25, 0.65, 0.10)
Very low (VL)	(0.15, 0.80, 0.05)
Extreme low (EL)	(0.05, 0.95, 0.00)

**Table 5:** Performance of alternatives according to the decision-maker.

Alternatives	C1	C2	C3	C4	C5	C6	C7	C8	C9	C10
A1	EH	EH	VH	M	L	MH	M	MH	M	VH
A2	EH	EH	H	M	M	H	M	M	MH	VH
A3	ML	M	MH	H	MH	EL	EL	M	H	MH
A4	ML	M	H	VH	H	M	L	M	EL	H
A5	EH	EH	VH	ML	L	VH	M	ML	H	H
A6	VL	L	EH	EL	VH	EH	VH	EL	H	MH
A7	L	MH	M	M	M	L	L	M	VH	M
A8	L	L	M	M	VH	L	EL	M	H	M
A9	EH	L	H	EL	L	MH	M	MH	M	H
A10	H	M	VH	M	L	H	VH	L	VL	H
A11	ML	MH	VH	M	L	H	VH	L	L	MH
A12	ML	MH	ML	ML	L	MH	VH	L	L	M
A13	EH	VH	H	MH	VH	L	ML	VH	MH	L
A14	EH	VH	VH	EL	H	VH	M	M	MH	MH
A15	EL	L	VH	ML	M	H	VH	ML	VH	ML
A16	L	L	EH	EL	MH	M	EH	ML	L	MH
A17	L	L	MH	VH	VH	ML	EL	MH	EH	H
A18	L	L	MH	M	M	H	H	ML	H	M
A19	L	L	MH	M	ML	H	H	ML	M	M
A20	VH	VH	VH	VH	VH	VH	M	M	H	VH

### Ethical approval and permission to participate

This article does not require ethical approval or permission to participate.

## Preliminary experiments and results

### Applying GIS

The development of the geographical information systems infrastructure and the introduction of new software connected to the systems have enabled employees to work in small-scale large areas. As a matter of fact, Erzurum, which is 25,355 km^2^ in size, has been handled at the provincial scale in the study. In the study, power lines and land use maps were obtained in the form of raster and these raster maps were transformed into a digital data set thanks to the Arc-GIS 10.2 package program (https://support.esri.com/en/products/desktop/arcgis-esktop/arcmap/10-2-2). Slope, aspect, solar radiation maps, which are among the other sources of the study, have been processed using the band data of the Landsat TM 8 satellite (https://earthexplorer.usgs.gov) Solar radiation maps based on the DEM data of the Landsat Tm 8 satellite. The solar radiation mapping that will fall on an average surface of the study area has been made. During the mapping process, the ArcGIS Spatial analyst toolbox solar radiation tools has been developed to calculate the insolation of the study area using the hemispherical field of view algorithm or for specific locations based on digital. Radar Topographic Mission (SRTM) 90 m × 90 m digital elevation model (DEM) was used in slope and aspect maps. In the study, surface temperature maps were used from Landsat TM8 satellite with 30 m spatial resolution band 4, band 5 and band 10 data (https://earthexplorer.usgs.gov). The air temperature and wind speed maps are obtained from the Global Solar Atlas and Global Wind Atlas digital platform as a digital model (www.globalsolaratlas.info/map) and adjusted to a scale suitable for the working area in GIS software. In addition, a different model was used to obtain the humidity and pressure maps used in the study. The data obtained from 11 different point stations belonging to the study area and its immediate surroundings were created by using the “Geostatistical Analyst” module in Arc GIS 10.2 software to create a polygon area covering the boundaries of the study area.

### Applying intuitionistic fuzzy approach to potential locations

This approach consists of six steps explained as follows:

**Step 1:** 10 Criteria are decided and the importance of each criterion is determined as shown in Table 3. These linguistic terms are converted into intuitionistic fuzzy sets. For example, the importance of C1 (Slope) is defined as “Important (I)” and its corresponding intuitionistic fuzzy number are (0.75, 0.20, 0.05).

**Step 2:** Performance of alternatives are evaluated using linguistic terms shown in Table 4. These terms are converted into intuitionistic fuzzy sets in the same way explained in Step 1.

**Step 3:** The aggregate intuitionistic fuzzy decision matrix for each alternative are calculated by multiplying the performance of each alternative by importance of each criterion.

**Step 4:** IFWAA operator is used to calculate the aggregate weighted intuitionistic fuzzy super decision matrix using Eq. (4).

**Step 5:** Intuitionistic fuzzy negative-ideal solution and positive-ideal solution are calculated as follows:

10 $${{\mathrm{\rm I}}^{+}}_{i}={(\mu }_{{{\mathrm{\rm I}}^{+}}_{i}}\left(x\right), {v}_{{{\mathrm{\rm I}}^{+}}_{i}}\left(x\right) , {\pi }_{{{\mathrm{\rm I}}^{+}}_{i}}\left(x\right) )$$

11 $${{\mathrm{\rm I}}^{-}}_{i}={(\mu }_{{{\mathrm{\rm I}}^{-}}_{i}}\left(x\right), {v}_{{{\mathrm{\rm I}}^{-}}_{i}}\left(x\right) , {\pi }_{{{\mathrm{\rm I}}^{-}}_{i}}\left(x\right) )$$

where ${{\mathrm{\rm I}}^{+}}_{i}$ represents an intuitionistic fuzzy positive ideal solution and ${{\mathrm{\rm I}}^{-}}_{i}$ indicates an intuitionistic fuzzy negative ideal solution.

12 $${\mu }_{{{\mathrm{ I}}^{+}}_{i}}\left(x\right)=\left(\underset{j}{{\max}}{{ \mu }}_{{\stackrel{\sim }{a}}_{{ij}}}\left(x\right)|\in {X}_{i}\right)$$

13 $${v}_{{{\mathrm{\rm I}}^{+}}_{i}}\left(x\right)=\left(\underset{j}{\mathrm{min}}{v}_{{\stackrel{\sim }{a}}_{\mathit{ij}}}\left(x\right)|\in {X}_{i}\right)$$

14 $${\mu }_{{{\mathrm{I}}^{-}}_{i}}\left(x\right)=\left(\underset{j}{\mathrm{min}}{{ \mu }}_{{\stackrel{\sim }{a}}_{\mathit{ij}}}\left(x\right)|\in {X}_{i}\right)$$

15 $${v}_{{{\mathrm{\rm I}}^{-}}_{i}}\left(x\right)=\left(\underset{j}{\mathrm{max}}{v}_{{\stackrel{\sim }{a}}_{\mathit{ij}}}\left(x\right)|\in {X}_{i}\right)$$

where ${X}_{i}$ is ith criterion and $\stackrel{\sim }{A}={[{\stackrel{\sim }{a}}_{ij}]}_{np}$ is the aggregate weighted intuitionistic fuzzy decision matrix where $i$ represents the number of criteria and $j$ indicates the number of alternatives.

**Step 6:** Positive and negative distances are measured using the intuitionistic fuzzy normalised Euclidean distance and results are seen Table 6.

**Table 6:** Positive and negative distances, closeness coefficients and the rank of alternatives.

Alternatives	Positive-ideal	Negative-ideal	Closeness coefficients	Rank
A1	0.2350	0.5986	0.7181	7
A2	0.2239	0.6133	0.7325	**5**
A3	0.3519	0.4874	0.5807	16
A4	0.2866	0.5533	0.6588	11
A5	0.2202	0.6136	0.7360	**4**
A6	0.1832	0.6409	0.7777	**2**
A7	0.4031	0.4342	0.5186	19
A8	0.3828	0.4530	0.5420	18
A9	0.3356	0.5020	0.5993	15
A10	0.2767	0.5627	0.6704	10
A11	0.3030	0.5354	0.6386	12
A12	0.4220	0.4158	0.4963	20
A13	0.2317	0.6047	0.7230	6
A14	0.2072	0.6281	0.7520	**3**
A15	0.2550	0.5803	0.6947	8
A16	0.3015	0.5257	0.6356	13
A17	0.2572	0.5770	0.6917	9
A18	0.3326	0.5090	0.6048	14
A19	0.3646	0.4764	0.5665	17
A20	0.1275	0.7087	0.8476	**1**

**Step 7:** The closeness coefficients are calculated based on negative-ideal solution and positive-ideal solution and the alternatives are ranked based on the closeness coefficients. The alternative with the highest value is chosen as the most suitable one.

## Experimental results

**Figure 16:**
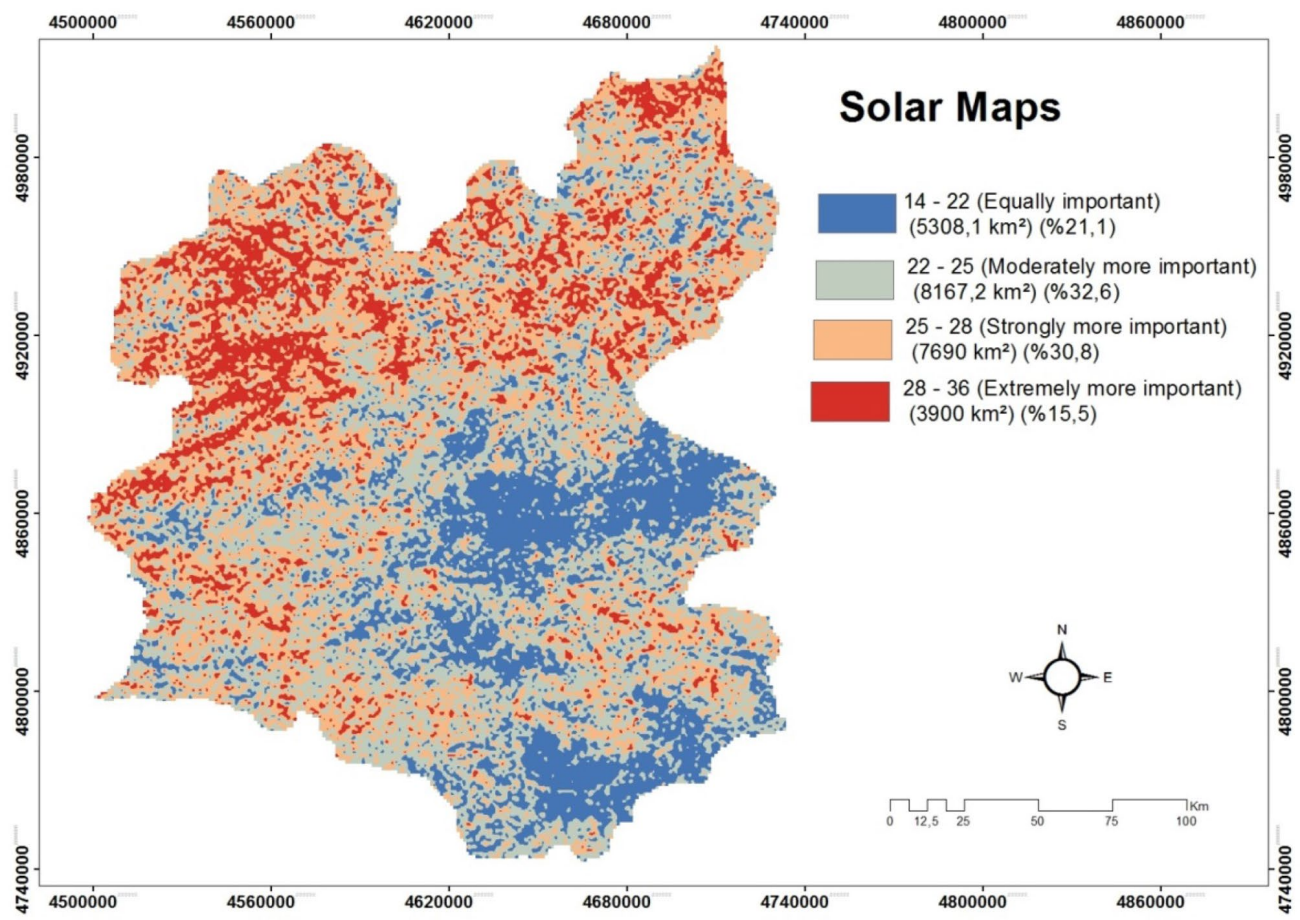
The solar maps of Erzurum.

The result obtained by GIS is shown in Fig. 16. According to the solar map created, it is found that 3900 km^2^ areas extremely more important, 7690 km^2^ strongly more important, 8167.2 km^2^moderately more important and 5308.1 km^2^ equally important for the solar site selection. Totally we found 25,065.3 km^2^ is the area that can be used for solar energy. We can obtain approximately 12,503,265 mW of Solar Energy from an area of 25,065.3 km^2^. In addition, the most of areas found as available for solar energy is located at between middle and the north sides of the city.

In intuitionistic fuzzy approach, alternatives are ranked as seen in Table 6 and based on this result, it is clearly seen that Yakutiye is the best place among 20 alternatives for solar power. The first five districts are found as Yakutiye, Ispir, Pasinler, Horasan and Aziziye. They all are located at between middle and the north sides of the city.

In addition, we compare GIS and intuitionistic fuzzy approaches and it is clearly seen that they both achieved the same area for the solar power plant installation. There are slightly different results found by the intuitionistic fuzzy approach. For example, the district “Olur” is located among the desirable areas defined by GIS while its rank is found as 20 as the worst one for the intuitionistic fuzzy approach. Due to its proximity to the Black Sea region, the climate in “Olur” district has changed to black sea climate from continental climate. This affects some important aspects negatively to install a solar power plant and the intuitionistic fuzzy approach as a multi-criteria decision making method considers all these aspects to cover the problem properly. In addition, “Tortum” is placed in 14th among 20 districts in the intuitionistic fuzzy approach while it is found as desirable area in GIS approach. The negative effect of geography makes it undesirable by the decision maker. To summarize, although, there are slightly different results achieved, the proposed intuitionistic fuzzy approach as a multi-criteria decision making method provides the same districts as the best option for solar power.

## Conclusion

Today, societies increase their competitive power, grow their economies, increase the quality of life, and are directly related to technological development levels. Technological developments can be achieved with sufficient, continuous and clean energy. The decrease in fossil energy resources and the environmental effects they cause have accelerated the shift towards renewable energy resources in energy supply. Among these energy resources, solar energy generation systems are considered environmentally friendly as they are free, continuous and renewable. Countries and international organizations apply high incentives to these technologies because solar energy is a clean energy source. In addition, the energy produced, regardless of its source, has different effects on the environment. In this context, when the environmental impacts caused by solar energy production systems are evaluated; it has been determined that solar power plants have a high level of land use effect, their carbon footprint and greenhouse gas effect is moderate, and the air pollution effect is very low. It has been observed that the effect of power plants on water resources is high in thermal solar systems, very low in photovoltaic solar systems, and local ecosystem, biodiversity and visual impact are high.

In developed countries, policies for the effective utilization of renewable energy and especially solar energy potential are put forward. However, while addressing these policies, it is aimed to minimize environmental impacts in renewable energy development processes. In this study, 20 districts of Erzurum are investigated in terms of 10 criteria (slope, aspect, solar irradiation, land use, wind speed, air temperature, air pressure, air humidity, land surface temperature and transmission line) using intuitionistic fuzzy sets. In these criteria are one of the parameters, the land surface temperature, is important. The sun-drenched land heats up fast, cools down quickly, gets very hot, becomes very cold. This affects the land surface temperature. The rapidly heated surface radiates heat faster and allows the solar panels to heat up. Heat and energy loss are high in the heated solar panel. In order for the energy loss not to be high, the temperature of the land surface area must be low. In this study, in addition to some criteria found in the literature in determining the most suitable location for a solar power plant, some criteria that are not used for a solar power plant in the literature, but are necessary. The importance values of the proposed new criteria and all subcriteria were found using the intuitionistic fuzzy method. In a sample study area, an application study was carried out in GIS environment with the help of digital data of the criteria. In the application, all digital data of the study area were processed according to the criterion weight values and combined in layers on top of each other, and the compatibility map in Fig. 16 was obtained for a possible solar power plant. On the suitability map of the area evaluated according to the applied parameters, the areas where the solar power plant installation is most suitable and suitable areas are revealed (25,065.3 km^2^ for solar energy Erzurum province has appropriate areas). With this study, it is aimed to contribute to the use of broader criteria in the solar power plant installation site determination studies in the literature using two different approaches. After defining the most suitable areas using GIS, 20 districts of Erzurum are evaluated along with the same criteria using an intuitionistic fuzzy approach. Considering the results obtained, the comparison of the two methods is made.Based on results found, it is clearly seen that two approaches reaches the same results for the solar power plant site selection problem defined. These results suggest that both approaches province of Erzurum in Turkey's one of the best locations for installation of solar power plants and under the parameters used in solar suitability maps were made in Erzurum provinces. Energy consumption cost analysis was made on the final compliance map.

There is a growing body of work on site selection problems for solar power plant installations and the proposed approach is insufficiently general and reusable. It can be applied to any site selection problem, ranging from renewable energy sources to agricultural area. As a future study, this approach can be developed considering more criteria in different applications in order not to ignore any criterion for site selection of the solar power plants installation.

## Data Availability

The data used in the publication were made from meteorology. It is widely mentioned in the “Material and method” section of the article.
